# Tumor analysis: freeze–thawing cycle of triple-negative breast cancer cells alters tumor CD24/CD44 profiles and the percentage of tumor-infiltrating immune cells

**DOI:** 10.1186/s13104-018-3504-5

**Published:** 2018-06-20

**Authors:** Matthieu Le Gallo, Thibault de la Motte Rouge, Amanda Poissonnier, Vincent Lavoué, Patrick Tas, Jean Leveque, Florence Godey, Patrick Legembre

**Affiliations:** 1 COSS (Chemistry Oncogenesis Stress Signaling)-UMR 1242, Inserm, Univ Rennes, CLCC Eugène Marquis, Rue Bataille Flandres Dunkerque, 35042 Rennes, France; 2 Equipe Labellisée Ligue Contre Le Cancer, Rue Bataille Flandres Dunkerque, 35042 Rennes, France; 3 CHU Pontchaillou, Rennes, France; 4 Present Address: Oregon Health and Science University, Portland, USA

**Keywords:** Triple negative breast cancer, Immune infiltrate, Living biobank, Frozen

## Abstract

**Objective:**

The use of novel methods to characterize living tumor cells relies on well-conceived biobanks. Herein, we raised the question of whether the composition of fresh and freeze/thawed dissociated tumor samples is comparable in terms of quantitative and qualitative profiling.

**Results:**

Breast cancer is a heterogeneous disease, encompassing luminal A and B, basal/triple-negative breast cancer (TNBC), and ERBB2-like tumors. We examined living cells dissociated from TNBC and found that a classical freeze/thaw protocol leads to a marked reduction in the number of CD45^−^CD44^Low^CD24^Low^ tumor cells. This, in turn, changed the percentage of tumor cells with certain CD44/CD24 expression patterns and changed the percentage of tumor-infiltrating immune cells. These cryopreservation-driven alterations in cellular phenotype make it impossible to compare fresh and frozen samples from the same patient directly. Moreover, the freeze/thaw process changed the transcriptomic signatures of triple-negative cancer stem cells in such a manner that hierarchical clustering no longer ranked them according to expected inter-individual differences. Overall, this study suggests that all analyses of living tumor cells should be conducted only using freshly dissociated tumors if we are to generate a robust scoring system for prognostic/predictive markers.

**Electronic supplementary material:**

The online version of this article (10.1186/s13104-018-3504-5) contains supplementary material, which is available to authorized users.

## Introduction

Personalized medicine necessitates identification of biomarkers that are accurate, sensitive, and disease-specific. This is particularly true for cancer, which is a disease characterized by marked tumor heterogeneity. Although thousands of biomarkers have been described, few have translated successfully to the clinic. Biomarkers are crucial not only for tumor diagnosis and prognosis, but also for better stratification of patients, which reduces concerns related to over-treatment of indolent cancers and under-treatment of aggressive cancers.

Among women, breast cancer is the most common cause of cancer, and the second leading cause of cancer death after lung cancer [1, 2]. Triple-negative breast cancer (TNBC) represents 15–20% of all breast cancer cases. It comprises a heterogeneous tumor subset that lacks expression of estrogen and progesterone receptors and does not overexpress HER2. As a group, TNBCs are aggressive and prognoses and clinical outcomes are poor [1]. The low percentage of tumor-infiltrating lymphocytes (TILs) [3] and accumulation of cancer stem cells (CSCs) [4] mean that TNBCs often show drug resistance, recurrence, and metastasis [5, 6]. Therefore, exhaustive characterization of tumor-infiltrating immune cells and tumor cell heterogeneity is crucial if we are to identify new prognostic and predictive biomarkers and therapeutic targets.

Most, if not all, genomic and proteomic studies are performed using flash-frozen tumor tissues; hopefully, such tissues will yield transcriptomic and/or protein signatures that can be used to develop a personalized medicine approach. However, the complexity and heterogeneity of TNBC tumors mean that clinicians have yet to achieve this goal. We hypothesized that the use of novel methods to exhaustively characterize dissociated and living tumor cells may move us a step closer. For instance, multiparameter flow cytometry (i.e., cyTOF, single cell sequencing) can reveal detailed signatures that are unique to cells inside a tumor (e.g., immune, stromal, and tumor cells) and, by so-doing, identify new markers associated with relapse, and/or targets for a new generation of therapeutic drugs. Accordingly, laboratories will come to rely on well-conceived biobanks to develop dissociated cancer tissues.

## Main text

### Methods and patients

#### Patients

Patients (n = 15) were diagnosed and treated at the Centre Eugène Marquis between 2017 and 2018. None showed evidence of relapse at the time of diagnosis and none received chemotherapy, endocrine therapy, or radiation therapy prior to surgery. Treatment decisions and follow-up processes were based solely on international recommendations.

#### Tumor samples

Triple-negative breast tumors were collected by a pathologist after resection by a surgeon and immediately placed in RPMI medium. The dissociation process initiated within 2 h after surgical resection. Tumors were dissociated using the tumor dissociation kit (Human) (Miltenyi Biotec GmbH), which is optimized to deliver a high yield of tumor cells and TILs while preserving important cell surface epitopes. TNBC pieces were weighed and cut into small pieces (< 2 mm^3^), which were then treated with dissociation kit (Human) in a gentleMACS Dissociator, according to the manufacturer’s recommendations. Briefly, tumors were mechanically dissociated in the gentleMACS dissociator for 36 s and then incubated at 37 °C for 30 min under continuous rotation. Next, a cycle of mechanical–chemical–mechanical dissociation was performed and dissociated cells were resuspended in RPMI. Macroscopic pieces were removed using a Corning^®^ cell strainer (70 μm). Tumor cells were then washed twice in RPMI (20 ml) and counted using a hemocytometer.

#### Flow cytometry

Tumor cells (50,000 cells) were suspended in PBS supplemented with 2% BSA, 2% FCS, and FcR block (Miltenyi Biotec GmbH) at 4 °C for 20 min. Cells were then stained for 30 min at 4 °C with anti-CD24 PE (clone ML5, BD Biosciences), anti-CD44 APC (clone G44-26, BD Biosciences), and anti-CD45 PE-Vio770 (clone 5B1, Miltenyi Biotec GmbH) antibodies. Isotypic antibodies were used as a control for each fluorochrome (obtained from the same manufacturers). Cells were then washed twice in PBS supplemented with 2% BSA and 2% FCS and resuspended in PBS. To assess cell viability, cells were incubated with 7-AAD (BD Biosciences) for 10 min prior to cytometry analysis. Data were acquired using a Novocyte cytometer (ACEA Biosciences) and analyzed using FlowJo or Novoexpress software.

#### Cryopreservation and storage

The freezing process was carried out using standardized freezing procedures, following the guidelines issued by the “Haute Autorité de Santé”, the government agency regulating the French healthcare system, for human tissue and cell samples biobanking. Freshly dissociated tumor cells were frozen in 1 ml of human serum albumin (HSA) Vialebex^®^ (LFB BIOMEDICAMENTS, Les Ullis, France) supplemented with 10% DMSO (Sigma Aldrich). Each vial contained 2–5 × 10^6^ cells (depending on the tumor dissociation yield). Briefly, freshly dissociated cell pellets were resuspended in 500 µl of pre-cooled HSA. Then, 500 µl of pre-cooled HSA containing 20% DMSO solution was added drop by drop to the cell suspension. The suspension was then homogenized and transferred to a cryotube. To ensure a standardized and controlled rate of freezing (− 1 °C/min), cryotubes were first placed in a CoolCell^®^ LX Cell Freezing Container (BioCision) at − 80 °C. After 24 h, cells were transferred to a freezer set at − 150 °C. All freezers were monitored; no critical temperature variations were recorded during storage.

For thawing, cells were placed in a water bath at 37 °C and then transferred to RPMI (40 ml) at RT to allow complete thawing. After a second wash in 20 ml of RPMI, the cells were counted using a hemocytometer. Cell viability was assessed using Trypan Blue.

#### Generation of mammospheres

Matched freshly dissociated and thawed tumor cells from the same patient were treated in the same way. Cells (1.5 × 10^6^) were seeded in 2 ml of Mammocult medium (StemCell Technologies) supplemented with heparin (4 µg/ml; Stem Cell Technologies), hydrocortisone (480 ng/ml; Stem Cell Technologies), penicillin (100 units/ml), and streptomycin (100 µg/ml) (Gibco) in ultra-low binding 6-well plates (Corning). After 15 days at 37 °C/5% CO_2_, mammospheres were collected and passed through a cell strainer (40 µm) to separate suspended cells from mammospheres. Next, mammospheres were dissociated with trypsin/EDTA (0.05% trypsin; Gibco) for 5 min. Dissociated cells were washed twice in PBS and stained as described above. RNA extracted from mammospheres using the NucleoSpin RNA XS extraction kit (Macherey–Nagel), according to the manufacturer’s recommendations.

#### Microarray analysis

RNA quality was assessed using an RNA6000 nano chip (Agilent). For each condition (fresh or freeze/thawed), 9 ng of RNA was reverse transcribed using the Ovation PicoSL WTA System V2 (Nugen, Leek, The Netherlands). Fragmented cDNAs were hybridized to GeneChip Human Gene 2.0 ST microarrays (Affymetrix), which were scanned by a GeneChip Scanner 3000 7G (Affymetrix). Raw data and quality-control metrics were generated using Expression Console software (Affymetrix). Probes were mapped using Brainarray V23 CDF files (http://brainarray.mbni.med.umich.edu/) and normalized by robust multi-array averaging with R software. Statistical analyses were performed using the limma R package; genes showing a twofold change in expression and a P value of 0.05 were considered significant. Gene Ontology terms enrichment analyses were performed using the ToppGene Suite (Chen J, Bardes EE, Aronow BJ, Jegga AG 2009. ToppGene Suite for gene list enrichment analysis and candidate gene prioritization. Nucleic Acids Research 10.1093/nar/gkp427). Hierarchical clustering was performed using Morpheus Matrix visualization and analysis software (https://software.broadinstitute.org/morpheus/).

#### Accession numbers

Raw and normalized data have been deposited to the GEO database accession ID GSE114359.

### Results

We raised the question of whether the composition of fresh and freeze/thawed samples from the same patients was sufficiently similar in terms of quantitative and qualitative profiling and asked whether both could be used to investigate TILs (CD45-positive cells) and the phenotype (CD45^neg^CD24^Low^CD44^High^) of CSCs. To address this, we used multiparameter flow cytometry to compare the phenotypes of 15 freshly dissociated TNBCs and their matched cryopreserved counterparts. Briefly, to minimize mortality due to sample processing time, the pathologist collected samples in RPMI medium and the dissociation process was started within a maximum of 120 min after surgical resection. Fresh TNBCs were dissociated using the Miltenyi Tumor dissociation kit (Human); 0.8 million cells were analyzed. In parallel, we cultured 1.5 × 10^6^ cells in serum-free medium in low-attachment plates to isolate spheroids, which are enriched in CSCs (see “Methods and patients” section). The remaining cells were frozen using a standardized procedure following the French “Haute Authorité de Santé” guidelines to biobanks and kept for 2 weeks in a freezer at − 150 °C. The cells were then thawed and analyzed immediately by flow cytometry; spheroids were also generated. Total RNA was isolated from fresh and thawed spheroids and the transcriptomic signatures compared (see Additional file 1: Figure S1).

**Fig. 1:**
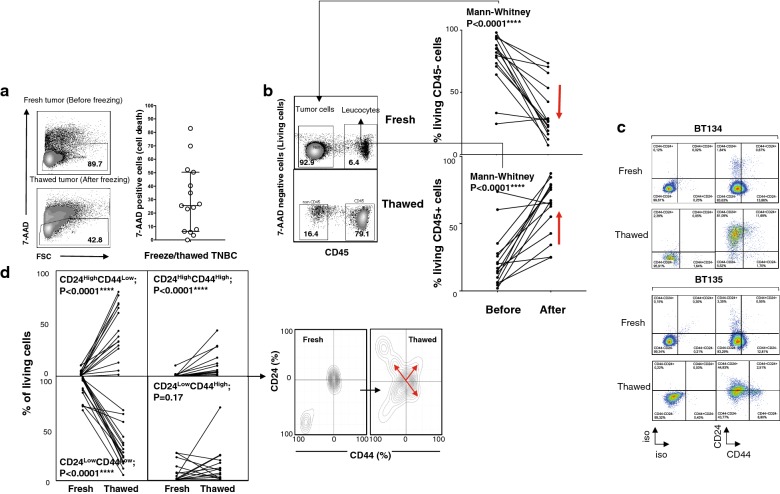
Flow cytometry analysis of freshly dissociated and frozen tumor samples. **a** *Left panels* viability of fresh (upper) or freeze/thawed (lower) tumor cells was assessed using the non-permeant fluorescent DNA marker 7-ADD. The gate shows the percentage of living cells. *Right panels* percentage of dead cells observed after thawing 15 TNBCs. **b** Leucocytes and non-immune cells (mainly tumor cells) within the dissociated tumor cell population were analyzed by flow cytometry after staining with an anti-CD45 mAb. The percentage of CD45+ (leukocytes) and CD45− (tumor cells) before and after freezing is depicted. Statistical differences were evaluated using the Mann–Whitney U test. **c** Two examples of fresh and freeze/thawed dissociated tumor cells. Expression of CD44 and CD24 by CD45− tumor cells was evaluated. **d** Comparison of the proportion of CD45− tumor cells showing CD24 and CD44 before and after the freeze/thaw process. P values were calculated using a paired Student’s *t* test (n = 15). Density plot showing the percentage of living tumor cells expressing CD24 and CD44 before and after cryopreservation

**Fig. 2:**
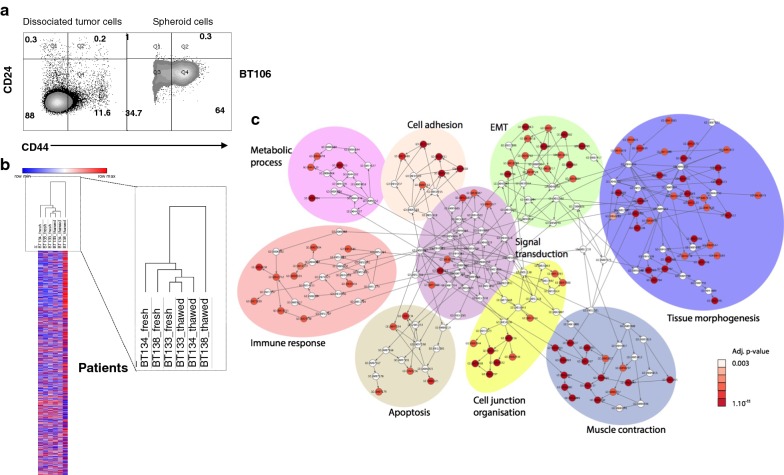
Transcriptomic signatures of fresh and freeze/thawed CSCs. **a** Flow cytometry analysis of freshly dissociated tumor cells and spheroids derived from the tumor after 15 days of culture in low serum/low adherence conditions. The dot plot is representative of data obtained from all 15 tumors. **b** Heatmap showing RNA expression by spheroids derived from fresh or frozen tumor cells from three different patients. Hierarchical clustering was performed on Robust Multi-Array Average (rma)-normalized expression data using the One minus Pearson’s correlation method. **c** Overall, 274 genes showed differential expression in spheroids isolated from fresh and frozen tumors. Network representation of GO terms (biological process) significantly enriched in the 224 genes downregulated in freeze/thawed mammospheres as compared to fresh ones. Similar go terms were spatially clustered and colors represent corrected p-values

Cell viability was assessed by 7-AAD staining. The results showed that the freezing/thawing procedure for dissociated TNBC cells yielded 25.4% dead cells, with five dissociated tumor samples showing a drop in absolute numbers of 10% or less, and four tumors showing more than 50% dead cells (Fig. 1a) after thawing. The reasons for this were unknown since all samples were treated in a similar manner. Strikingly, most of the cells that died following the freezing/thawing process originated from the CD45− population (Fig. 1b), which in turn impacted the percentage of tumor-infiltrating leukocytes (i.e., CD45+ cells; Fig. 1b). Of note, the percentage of tumor cells with a CD45^−^CD24^Low^CD44^Low^ phenotype fell markedly after thawing (Fig. 1c, d). Two hypotheses may explain these losses: either these cells are highly sensitive to freezing/thawing, or they are reprogrammed to exhibit a different CD24/CD44 phenotype. The percentage of dead cells observed in Fig. 1a and the short delay between thawing and immunophenotyping are not compatible with the latter hypothesis, strongly suggesting that CD45^−^CD24^Low^CD44^Low^ cells do not tolerate the freeze/thaw process well. Loss of this population resulted mainly in artificial enrichment of the CD24^High^CD44^Low^ cell population and, albeit to a lesser extent, the CD24^Low^CD44^High^ and CD24^High^CD44^High^ populations (Fig. 1d). Because the CD24^Low^CD44^High^ lineage was enriched after the freeze/thaw cycle, and this cell subset seems to correspond to stem/progenitor cancer cells [5, 6], we next wondered whether the transcriptomic signature of these enriched CSCs varied after the freezing/thawing process. To compare the transcriptome of CSCs derived from fresh and frozen samples, we used stringent cell culture procedures to culture spheroid cells. Compared with the bulk of dissociated tumor cells, spheroids were enriched for CD24^Low^CD44^High^ cells (Fig. 2a). Next, we examined freshly dissociated TNBC cells from three different TNBC patients, along with the cells from the same patients frozen immediately after dissociation and cryopreserved for 15 days before thawing. Both sets of cells were cultured for 15 days in serum-free medium in low-adherence plates to select CSCs (CD44^High^CD24^Low^) and the transcriptomic signatures were compared (Fig. 2b). Unexpectedly, unsupervised hierarchical clustering of these tumor cells ranked tumors according to whether they had been subjected to freeze/thaw treatment and not according to their inter-individual differences; this shows that freeze/thawing the cells had a deleterious effect in their transcriptomic signatures. Gene Ontology terms enrichment analysis revealed that the freeze/thaw process drove deregulation of 274 genes (-fold change > 2, P < 0.05); most of these were associated to epithelial to mesenchymal transition (EMT), tissues morphogenesis and cell junction and adhesion (Fig. 2c). Of these, 224 genes (82%) in freeze/thaw spheroids were significantly down-regulated when compared with those in fresh spheroids( Table 1). Because these genes play a role in organization of cell junctions and are lost during freeze/thaw of CSCs, we suggest that cryopreservation either spares breast cancer stem cells showing the most dedifferentiated mesenchymal profiles or favors EMT reprogramming (Fig. 2c and Additional file 2: Table S1).

**Table 1:** Transcriptomic comparison between fresh TNBC cells and their freeze/thawed counterparts

ProbeId	Gene name	logFC	FC	t	P value	Adj. P val	B	DESC	ENTREZID	ENSEMBL	BT133_fresh	BT134_fresh	BT138_fresh	BT133_thawed	BT134_thawed	BT138_thawed
10216_at	PRG4	2.97	7.81	2.57	0.04	0.85	− 4.41	Proteoglycan 4	10216.00	ENSG00000116690	5.18	3.84	5.88	8.73	5.41	9.64
664613_at	MIR544A	2.46	5.48	4.28	0.00	0.85	− 4.29	MicroRNA 544a	664613.00	ENSG00000207587	4.13	2.41	2.94	4.70	6.14	6.01
767568_at	SNORD113-8	2.42	5.35	2.56	0.04	0.85	− 4.41	Small nucleolar RNA, C/D box 113-8	767568.00	ENSG00000200367	3.96	3.09	3.01	4.14	5.27	7.91
407000_at	MIR218-1	2.31	4.95	2.71	0.03	0.85	− 4.40	MicroRNA 218-1	407000.00	ENSG00000207732	4.05	3.62	3.13	4.19	5.86	7.67
102723604_at	LOC102723604	2.14	4.42	3.04	0.02	0.85	− 4.37	Uncharacterized LOC102723604	102723604.00	ENSG00000258928	5.04	3.47	5.05	5.37	6.98	7.63
105369519_at	LOC105369519	2.08	4.22	2.52	0.04	0.85	− 4.42	Uncharacterized LOC105369519	105369519.00	NA	2.96	3.41	3.46	3.78	5.09	7.19
2633_at	GBP1	2.06	4.17	2.62	0.04	0.85	− 4.41	Guanylate binding protein 1	2633.00	ENSG00000117228	5.85	3.16	3.90	6.79	6.98	5.32
26822_at	SNORD14A	2.05	4.13	3.54	0.01	0.85	− 4.33	Small nucleolar RNA, C/D box 14A	26822.00	ENSG00000272034	5.95	3.94	4.02	6.68	7.05	6.30
767579_at	SNORD114-3	1.99	3.97	2.87	0.03	0.85	− 4.38	Small nucleolar RNA, C/D box 114-3	767579.00	ENSG00000201839	4.05	2.30	2.54	5.67	5.51	3.68
342908_at	ZNF404	1.78	3.44	4.01	0.01	0.85	− 4.30	Zinc finger protein 404	342908.00	ENSG00000176222	5.56	5.29	4.22	7.18	6.18	7.07
107983995_at	NA	1.65	3.14	3.44	0.01	0.85	− 4.34	NA	NA	NA	3.95	3.77	3.77	5.11	4.74	6.58
767606_at	SNORD114-26	1.64	3.11	3.43	0.01	0.85	− 4.34	Small nucleolar RNA, C/D box 114-26	767606.00	ENSG00000200413	3.98	2.44	2.47	5.02	4.20	4.59
664612_at	MIR539	1.57	2.98	3.38	0.01	0.85	− 4.34	MicroRNA 539	664612.00	ENSG00000202560	7.15	7.17	7.22	7.77	8.84	9.66
54518_at	APBB1IP	1.57	2.97	2.52	0.04	0.85	− 4.42	Amyloid beta precursor protein binding family B member 1 interacting protein	54518.00	ENSG00000077420	6.12	3.54	4.64	6.29	6.31	6.39
100033820_at	SNORD116-28	1.53	2.88	2.46	0.05	0.85	− 4.42	Small nucleolar RNA, C/D box 116-28	100033820.00	ENSG00000278123	4.08	2.25	3.17	4.17	4.15	5.75
8404_at	SPARCL1	1.48	2.78	2.65	0.04	0.85	− 4.40	SPARC like 1	8404.00	ENSG00000152583	6.89	4.74	5.71	7.73	7.09	6.94
100289635_at	ZNF605	1.45	2.73	3.65	0.01	0.85	− 4.32	Zinc finger protein 605	100289635.00	ENSG00000196458	6.09	4.60	5.45	7.06	6.56	6.86
105376805_at	LOC105376805	1.41	2.66	3.09	0.02	0.85	− 4.36	Uncharacterized LOC105376805	105376805.00	ENSG00000238142	4.47	4.24	3.96	4.62	6.27	6.01
100128002_at	LOC100128002	1.41	2.66	2.52	0.04	0.85	− 4.42	Uncharacterized LOC100128002	100128002.00	NA	3.36	3.72	3.03	4.18	6.05	4.11
158131_at	OR1Q1	1.36	2.57	2.49	0.05	0.85	− 4.42	Olfactory receptor family 1 subfamily Q member 1	158131.00	ENSG00000165202	3.88	2.84	3.53	5.66	3.69	5.00
100616251_at	MIR1587	1.36	2.57	2.60	0.04	0.85	− 4.41	MicroRNA 1587	100616251.00	ENSG00000263972	4.55	4.60	4.25	5.02	5.44	7.03
106481206_at	RNU6-94P	1.31	2.47	2.59	0.04	0.85	− 4.41	RNA, U6 small nuclear 94, pseudogene	106481206.00	NA	3.97	3.61	3.31	5.37	3.82	5.62
57562_at	CEP126	1.30	2.46	4.04	0.01	0.85	− 4.30	Centrosomal protein 126	57562.00	ENSG00000110318	4.80	3.93	4.71	6.19	5.70	5.45
5170_at	PDPK1	1.29	2.44	2.47	0.05	0.85	− 4.42	3-Phosphoinositide dependent protein kinase 1	5170.00	ENSG00000140992	4.17	3.49	3.67	6.09	5.04	4.07
26787_at	SNORD61	1.28	2.43	3.60	0.01	0.85	− 4.33	Small nucleolar RNA, C/D box 61	26787.00	ENSG00000206979	2.63	2.18	3.34	3.57	4.13	4.28
26770_at	SNORD79	1.23	2.35	2.91	0.03	0.85	− 4.38	Small nucleolar RNA, C/D box 79	26770.00	NA	5.33	4.32	5.01	6.87	5.94	5.55
90499_at	FAM95A	1.22	2.32	2.49	0.05	0.85	− 4.42	Family with sequence similarity 95 member A	90499.00	NA	3.93	3.53	3.65	4.23	4.49	6.04
100126355_at	MIR365A	1.20	2.30	2.60	0.04	0.85	− 4.41	MicroRNA 365a	100126355.00	ENSG00000199130	5.26	3.96	4.05	6.12	4.97	5.78
441728_at	LOC441728	1.20	2.29	3.29	0.02	0.85	− 4.35	Golgin-like pseudogene	441728.00	NA	3.29	3.39	3.15	4.38	3.82	5.21
5228_at	PGF	1.19	2.28	2.78	0.03	0.85	− 4.39	Placental growth factor	5228.00	ENSG00000119630	3.93	3.80	5.04	4.97	5.99	5.36
619564_at	SNORD72	1.17	2.24	3.08	0.02	0.85	− 4.36	Small nucleolar RNA, C/D box 72	619564.00	ENSG00000212296	2.29	1.80	1.89	2.94	3.95	2.60
168667_at	BMPER	1.15	2.21	5.79	0.00	0.85	− 4.24	BMP binding endothelial regulator	168667.00	ENSG00000164619	4.90	4.78	4.60	6.05	5.64	6.02
64853_at	AIDA	1.15	2.21	2.51	0.05	0.85	− 4.42	Axin interactor, dorsalization associated	64853.00	ENSG00000186063	4.41	2.58	3.68	4.84	4.63	4.64
100462981_at	MTRNR2L2	1.15	2.21	2.64	0.04	0.85	− 4.40	MT-RNR2-like 2	100462981.00	ENSG00000269028	2.95	2.61	3.15	3.89	3.31	4.95
100379296_at	RNY4P13	1.14	2.20	3.05	0.02	0.85	− 4.37	RNA, Ro-associated Y4 pseudogene 13	100379296.00	NA	3.42	3.02	3.47	4.06	4.02	5.24
100873920_at	NHS-AS1	1.12	2.17	4.52	0.00	0.85	− 4.28	NHS antisense RNA 1	100873920.00	ENSG00000230020	3.29	2.94	3.06	3.81	4.57	4.27
340527_at	NHSL2	1.11	2.16	3.27	0.02	0.85	− 4.35	NHS like 2	340527.00	ENSG00000204131	4.91	4.01	4.04	5.86	5.05	5.38
7857_at	SCG2	1.10	2.15	3.01	0.02	0.85	− 4.37	Secretogranin II	7857.00	ENSG00000171951	4.11	3.41	4.48	5.64	4.83	4.84
503582_at	ARGFX	1.10	2.14	2.84	0.03	0.85	− 4.39	Arginine-fifty homeobox	503582.00	ENSG00000186103	2.98	2.69	2.91	3.90	3.24	4.74
574494_at	MIR521-1	1.10	2.14	2.91	0.03	0.85	− 4.38	MicroRNA 521-1	574494.00	ENSG00000207634	3.23	3.03	3.58	3.92	4.03	5.17
26022_at	TMEM98	1.08	2.12	4.33	0.00	0.85	− 4.29	Transmembrane protein 98	26022.00	ENSG00000006042	4.10	3.42	3.55	4.73	4.57	5.03
343521_at	TCTEX1D4	1.08	2.11	5.33	0.00	0.85	− 4.25	Tctex1 domain containing 4	343521.00	ENSG00000188396	4.98	4.86	4.69	5.83	6.19	5.73
4919_at	ROR1	1.07	2.10	2.78	0.03	0.85	− 4.39	Receptor tyrosine kinase like orphan receptor 1	4919.00	ENSG00000185483	4.27	4.19	5.12	6.24	5.25	5.30
440910_at	LOC440910	1.07	2.09	5.58	0.00	0.85	− 4.24	Uncharacterized LOC440910	440910.00	ENSG00000231431	4.11	3.73	3.77	5.05	4.78	4.98
105373893_at	LOC105373893	1.06	2.09	3.87	0.01	0.85	− 4.31	Uncharacterized LOC105373893	105373893.00	NA	2.54	3.11	3.03	3.60	4.36	3.91
105375249_at	LOC105375249	1.06	2.08	2.92	0.03	0.85	− 4.38	Uncharacterized LOC105375249	105375249.00	NA	3.06	2.60	2.85	3.12	4.33	4.23
84973_at	SNHG7	1.03	2.05	2.97	0.02	0.85	− 4.37	Small nucleolar RNA host gene 7	84973.00	ENSG00000233016	4.84	4.29	4.02	5.62	4.82	5.81
400728_at	FAM87B	1.03	2.04	3.33	0.02	0.85	− 4.35	Family with sequence similarity 87 member B	400728.00	ENSG00000177757	4.65	4.11	4.01	5.48	4.76	5.63
283685_at	GOLGA6L2	1.03	2.04	2.49	0.05	0.85	− 4.42	Golgin A6 family-like 2	283685.00	ENSG00000174450	4.22	4.06	4.06	4.52	4.84	6.07
9376_at	SLC22A8	1.01	2.02	3.32	0.02	0.85	− 4.35	Solute carrier family 22 member 8	9376.00	ENSG00000149452	3.30	3.43	3.29	4.09	3.97	5.00
9787_at	DLGAP5	− 1.00	− 2.00	− 2.66	0.04	0.85	− 4.40	DLG associated protein 5	9787.00	ENSG00000126787	5.91	5.43	4.60	4.59	4.40	3.95
5709_at	PSMD3	− 1.00	− 2.00	− 3.12	0.02	0.85	− 4.36	Proteasome 26S subunit, non-ATPase 3	5709.00	ENSG00000108344	6.23	6.90	7.03	5.88	6.05	5.23
5154_at	PDGFA	− 1.00	− 2.00	− 3.42	0.01	0.85	− 4.34	Platelet derived growth factor subunit A	5154.00	ENSG00000197461	5.01	6.02	5.81	4.54	4.60	4.69
163050_at	ZNF564	− 1.00	− 2.00	− 3.74	0.01	0.85	− 4.32	Zinc finger protein 564	163050.00	ENSG00000249709	5.69	6.30	6.20	4.67	5.30	5.22
7405_at	UVRAG	− 1.00	− 2.01	− 2.59	0.04	0.85	− 4.41	UV radiation resistance associated	7405.00	ENSG00000198382	6.49	7.01	7.35	6.44	6.17	5.23
283624_at	LINC00641	− 1.01	− 2.01	− 3.15	0.02	0.85	− 4.36	Long intergenic non-protein coding RNA 641	283624.00	ENSG00000258441	5.99	6.25	6.36	5.41	5.63	4.55
3931_at	LCAT	− 1.01	− 2.01	− 5.27	0.00	0.85	− 4.25	Lecithin-cholesterol acyltransferase	3931.00	ENSG00000213398	5.49	5.73	5.80	4.83	4.71	4.45
1318_at	SLC31A2	− 1.01	− 2.01	− 2.96	0.02	0.85	− 4.38	Solute carrier family 31 member 2	1318.00	ENSG00000136867	5.63	6.51	6.83	5.56	5.17	5.21
4771_at	NF2	− 1.01	− 2.02	− 2.70	0.03	0.85	− 4.40	Neurofibromin 2	4771.00	ENSG00000186575	5.54	6.07	5.83	5.22	5.18	4.00
54947_at	LPCAT2	− 1.01	− 2.02	− 2.51	0.05	0.85	− 4.42	Lysophosphatidylcholine acyltransferase 2	54947.00	ENSG00000087253	5.56	6.63	7.04	5.19	5.34	5.67
1978_at	EIF4EBP1	− 1.01	− 2.02	− 2.98	0.02	0.85	− 4.37	Eukaryotic translation initiation factor 4E binding protein 1	1978.00	ENSG00000187840	5.94	6.91	6.67	5.68	5.79	5.01
407043_at	MIR7-1	− 1.02	− 2.02	− 3.21	0.02	0.85	− 4.36	MicroRNA 7-1	407043.00	ENSG00000284179	4.77	5.46	5.67	4.54	3.86	4.46
8893_at	EIF2B5	− 1.02	− 2.03	− 2.44	0.05	0.85	− 4.42	Eukaryotic translation initiation factor 2B subunit epsilon	8893.00	ENSG00000145191	6.73	7.46	6.76	6.48	6.28	5.13
205564_at	SENP5	− 1.02	− 2.03	− 2.45	0.05	0.85	− 4.42	SUMO1/sentrin specific peptidase 5	205564.00	ENSG00000119231	6.72	6.75	7.15	6.53	6.06	4.98
2305_at	FOXM1	− 1.02	− 2.03	− 2.84	0.03	0.85	− 4.39	Forkhead box M1	2305.00	ENSG00000111206	5.61	5.60	5.15	4.64	4.96	3.71
113263_at	GLCCI1	− 1.02	− 2.03	− 2.65	0.04	0.85	− 4.40	Glucocorticoid induced 1	113263.00	ENSG00000106415	6.15	6.57	6.53	6.11	5.41	4.66
2030_at	SLC29A1	− 1.03	− 2.04	− 2.60	0.04	0.85	− 4.41	Solute carrier family 29 member 1 (Augustine blood group)	2030.00	ENSG00000112759	5.22	6.50	6.64	5.02	5.06	5.20
6660_at	SOX5	− 1.03	− 2.04	− 4.88	0.00	0.85	− 4.27	SRY-box 5	6660.00	ENSG00000134532	4.32	3.94	4.54	3.13	3.33	3.24
144100_at	PLEKHA7	− 1.03	− 2.04	− 2.57	0.04	0.85	− 4.41	Pleckstrin homology domain containing A7	144100.00	ENSG00000166689	3.85	5.38	4.81	3.67	3.85	3.44
23248_at	RPRD2	− 1.03	− 2.04	− 2.45	0.05	0.85	− 4.42	Regulation of nuclear pre-mRNA domain containing 2	23248.00	ENSG00000163125	6.94	7.36	7.59	6.80	6.62	5.38
26273_at	FBXO3	− 1.03	− 2.05	− 3.22	0.02	0.85	− 4.35	F-box protein 3	26273.00	ENSG00000110429	5.28	6.10	6.40	5.06	4.69	4.94
57037_at	ANKMY2	− 1.03	− 2.05	− 2.84	0.03	0.85	− 4.39	Ankyrin repeat and MYND domain containing 2	57037.00	ENSG00000106524	5.45	6.12	5.33	5.19	4.55	4.06
9701_at	PPP6R2	− 1.04	− 2.05	− 2.88	0.03	0.85	− 4.38	Protein phosphatase 6 regulatory subunit 2	9701.00	ENSG00000100239	5.03	5.51	5.22	4.64	4.56	3.46
10564_at	ARFGEF2	− 1.04	− 2.05	− 2.46	0.05	0.85	− 4.42	ADP ribosylation factor guanine nucleotide exchange factor 2	10564.00	ENSG00000124198	7.10	7.70	7.10	6.75	6.66	5.37
79005_at	SCNM1	− 1.04	− 2.06	− 3.37	0.01	0.85	− 4.34	Sodium channel modifier 1	79005.00	ENSG00000163156	4.53	4.80	5.40	4.27	3.81	3.54
6558_at	SLC12A2	− 1.04	− 2.06	− 2.88	0.03	0.85	− 4.38	Solute carrier family 12 member 2	6558.00	ENSG00000064651	7.47	8.24	7.42	7.15	6.77	6.09
377677_at	CA13	− 1.05	− 2.06	− 3.02	0.02	0.85	− 4.37	Carbonic anhydrase 13	377677.00	ENSG00000185015	3.82	4.88	4.77	3.82	3.32	3.19
10678_at	B3GNT2	− 1.05	− 2.07	− 5.23	0.00	0.85	− 4.25	UDP-GlcNAc:betaGal beta-1,3-N-acetylglucosaminyltransferase 2	10678.00	ENSG00000170340	6.95	7.17	7.39	6.29	5.94	6.15
6641_at	SNTB1	− 1.05	− 2.07	− 2.91	0.03	0.85	− 4.38	syntrophin beta 1	6641.00	ENSG00000172164	6.23	7.02	7.18	5.99	6.09	5.19
51050_at	PI15	− 1.06	− 2.08	− 3.28	0.02	0.85	− 4.35	Peptidase inhibitor 15	51050.00	ENSG00000137558	4.70	3.74	3.81	2.77	2.96	3.35
222068_at	TMED4	− 1.06	− 2.08	− 2.62	0.04	0.85	− 4.41	Transmembrane p24 trafficking protein 4	222068.00	ENSG00000158604	6.22	6.92	6.84	6.17	5.82	4.81
1719_at	DHFR	− 1.06	− 2.08	− 2.82	0.03	0.85	− 4.39	Dihydrofolate reductase	1719.00	ENSG00000228716	4.72	5.34	5.88	4.73	4.22	3.81
51203_at	NUSAP1	− 1.06	− 2.09	− 2.70	0.03	0.85	− 4.40	Nucleolar and spindle associated protein 1	51203.00	ENSG00000137804	6.15	5.52	5.25	4.53	5.23	3.97
6217_at	RPS16	− 1.06	− 2.09	− 2.75	0.03	0.85	− 4.39	Ribosomal protein S16	6217.00	ENSG00000105193	8.53	9.00	9.28	8.50	7.94	7.17
28960_at	DCPS	− 1.07	− 2.10	− 2.51	0.05	0.85	− 4.42	Decapping enzyme, scavenger	28960.00	ENSG00000110063	5.10	6.58	5.59	4.75	5.06	4.25
9929_at	JOSD1	− 1.07	− 2.10	− 2.52	0.04	0.85	− 4.42	Josephin domain containing 1	9929.00	ENSG00000100221	6.81	7.41	7.05	6.93	5.64	5.48
54534_at	MRPL50	− 1.07	− 2.10	− 2.90	0.03	0.85	− 4.38	Mitochondrial ribosomal protein L50	54534.00	ENSG00000136897	5.55	6.48	6.67	5.08	5.61	4.80
51706_at	CYB5R1	− 1.08	− 2.11	− 2.67	0.04	0.85	− 4.40	Cytochrome b5 reductase 1	51706.00	ENSG00000159348	5.42	5.82	6.36	4.62	5.51	4.24
6615_at	SNAI1	− 1.08	− 2.11	− 4.82	0.00	0.85	− 4.27	Snail family transcriptional repressor 1	6615.00	ENSG00000124216	5.55	5.85	5.80	4.36	4.62	4.99
1063_at	CENPF	− 1.08	− 2.12	− 3.03	0.02	0.85	− 4.37	Centromere protein F	1063.00	ENSG00000117724	7.15	6.27	6.93	5.82	6.15	5.12
5832_at	ALDH18A1	− 1.08	− 2.12	− 2.73	0.03	0.85	− 4.40	Aldehyde dehydrogenase 18 family member A1	5832.00	ENSG00000059573	6.94	7.26	7.20	6.51	6.48	5.16
116092_at	DNTTIP1	− 1.09	− 2.12	− 2.57	0.04	0.85	− 4.41	Deoxynucleotidyltransferase terminal interacting protein 1	116092.00	ENSG00000101457	5.89	6.68	6.59	5.61	5.82	4.47
6241_at	RRM2	− 1.10	− 2.14	− 2.83	0.03	0.85	− 4.39	Ribonucleotide reductase regulatory subunit M2	6241.00	ENSG00000171848	7.05	6.78	5.62	5.47	5.24	5.44
54764_at	ZRANB1	− 1.10	− 2.14	− 3.01	0.02	0.85	− 4.37	Zinc finger RANBP2-type containing 1	54764.00	ENSG00000019995	7.41	8.66	7.86	7.18	6.91	6.54
50_at	ACO2	− 1.10	− 2.14	− 2.43	0.05	0.85	− 4.43	Aconitase 2	50.00	ENSG00000100412	4.95	6.14	5.36	4.01	5.18	3.97
84546_at	SNORD35B	− 1.10	− 2.15	− 2.56	0.04	0.85	− 4.41	Small nucleolar RNA, C/D box 35B	84546.00	ENSG00000200530	5.69	5.74	6.57	4.42	5.71	4.55
677845_at	SNORA79	− 1.11	− 2.15	− 2.50	0.05	0.85	− 4.42	Small nucleolar RNA, H/ACA box 79	677845.00	ENSG00000221303	4.99	6.70	5.51	4.76	4.70	4.42
54700_at	RRN3	− 1.11	− 2.16	− 2.52	0.04	0.85	− 4.42	RRN3 homolog, RNA polymerase I transcription factor	54700.00	ENSG00000085721, ENSG00000278494	5.79	5.48	5.91	5.61	4.19	4.05
56907_at	SPIRE1	− 1.11	− 2.17	− 3.21	0.02	0.85	− 4.35	Spire type actin nucleation factor 1	56907.00	ENSG00000134278	6.98	7.96	7.50	6.63	6.63	5.83
1739_at	DLG1	− 1.11	− 2.17	− 3.42	0.01	0.85	− 4.34	Discs large MAGUK scaffold protein 1	1739.00	ENSG00000075711	6.80	7.23	7.18	6.51	6.01	5.36
9991_at	PTBP3	− 1.12	− 2.17	− 2.96	0.02	0.85	− 4.38	Polypyrimidine tract binding protein 3	9991.00	ENSG00000119314	8.29	9.03	8.69	7.97	7.88	6.81
54512_at	EXOSC4	− 1.12	− 2.17	− 2.89	0.03	0.85	− 4.38	Exosome component 4	54512.00	ENSG00000178896	6.25	7.39	6.94	5.57	6.31	5.35
56910_at	STARD7	− 1.12	− 2.17	− 2.79	0.03	0.85	− 4.39	StAR related lipid transfer domain containing 7	56910.00	ENSG00000084090	8.17	8.42	9.00	7.59	7.98	6.66
5894_at	RAF1	− 1.12	− 2.17	− 2.62	0.04	0.85	− 4.41	Raf-1 proto-oncogene, serine/threonine kinase	5894.00	ENSG00000132155	7.08	7.18	7.55	6.44	6.79	5.22
3099_at	HK2	− 1.12	− 2.18	− 2.80	0.03	0.85	− 4.39	Hexokinase 2	3099.00	ENSG00000159399	6.52	7.97	7.18	6.28	6.31	5.72
1017_at	CDK2	− 1.13	− 2.18	− 2.60	0.04	0.85	− 4.41	Cyclin dependent kinase 2	1017.00	ENSG00000123374	6.47	7.67	7.78	5.95	6.73	5.86
9388_at	LIPG	− 1.13	− 2.19	− 2.82	0.03	0.85	− 4.39	Lipase G, endothelial type	9388.00	ENSG00000101670	4.66	6.09	5.53	4.62	4.32	3.94
100128398_at	LOC100128398	− 1.14	− 2.20	− 2.82	0.03	0.85	− 4.39	Uncharacterized LOC100128398	100128398.00	ENSG00000176593	4.70	4.73	5.99	4.23	4.13	3.65
8714_at	ABCC3	− 1.14	− 2.21	− 2.45	0.05	0.85	− 4.42	ATP binding cassette subfamily C member 3	8714.00	ENSG00000108846	6.34	7.36	7.00	6.03	6.39	4.86
64866_at	CDCP1	− 1.15	− 2.22	− 2.46	0.05	0.85	− 4.42	CUB domain containing protein 1	64866.00	ENSG00000163814	7.31	8.23	7.10	7.00	6.62	5.57
2673_at	GFPT1	− 1.15	− 2.23	− 2.86	0.03	0.85	− 4.38	Glutamine--fructose-6-phosphate transaminase 1	2673.00	ENSG00000198380	7.65	8.48	7.99	7.48	7.05	6.13
6376_at	CX3CL1	− 1.16	− 2.23	− 2.48	0.05	0.85	− 4.42	C-X3-C motif chemokine ligand 1	6376.00	ENSG00000006210	5.36	6.55	6.82	4.74	5.71	4.81
116113_at	FOXP4	− 1.16	− 2.24	− 3.51	0.01	0.85	− 4.33	Forkhead box P4	116113.00	ENSG00000137166	5.23	6.44	5.95	4.53	4.84	4.78
203413_at	CT83	− 1.16	− 2.24	− 2.49	0.05	0.85	− 4.42	Cancer/testis antigen 83	203413.00	ENSG00000204019	4.95	6.72	5.25	4.50	4.48	4.45
8503_at	PIK3R3	− 1.17	− 2.25	− 2.52	0.04	0.85	− 4.42	Phosphoinositide-3-kinase regulatory subunit 3	8503.00	ENSG00000117461, ENSG00000278139	6.13	7.36	5.60	5.48	5.17	4.93
22992_at	KDM2A	− 1.17	− 2.25	− 2.51	0.04	0.85	− 4.42	Lysine demethylase 2A	22992.00	ENSG00000173120	8.13	8.42	8.27	7.76	7.52	6.03
55707_at	NECAP2	− 1.18	− 2.26	− 2.77	0.03	0.85	− 4.39	NECAP endocytosis associated 2	55707.00	ENSG00000157191	5.93	6.96	7.07	5.21	6.14	5.08
55227_at	LRRC1	− 1.18	− 2.26	− 2.74	0.03	0.85	− 4.39	Leucine rich repeat containing 1	55227.00	ENSG00000137269	3.60	5.28	4.36	3.44	3.21	3.05
8694_at	DGAT1	− 1.19	− 2.28	− 2.63	0.04	0.85	− 4.40	Diacylglycerol *O*-acyltransferase 1	8694.00	ENSG00000185000	5.46	6.59	7.05	5.26	5.54	4.74
64225_at	ATL2	− 1.19	− 2.28	− 3.39	0.01	0.85	− 4.34	Atlastin GTPase 2	64225.00	ENSG00000119787	5.73	6.75	6.41	5.31	5.42	4.61
6301_at	SARS	− 1.19	− 2.28	− 2.62	0.04	0.85	− 4.41	Seryl-tRNA synthetase	6301.00	ENSG00000031698	6.58	6.90	7.30	6.15	6.30	4.76
6536_at	SLC6A9	− 1.19	− 2.28	− 3.92	0.01	0.85	− 4.31	Solute carrier family 6 member 9	6536.00	ENSG00000196517	6.25	6.41	7.04	5.30	5.04	5.79
4494_at	MT1F	− 1.19	− 2.29	− 2.61	0.04	0.85	− 4.41	Metallothionein 1F	4494.00	ENSG00000198417	7.00	7.65	7.47	6.32	6.97	5.25
54069_at	MIS18A	− 1.20	− 2.29	− 3.11	0.02	0.85	− 4.36	MIS18 kinetochore protein A	54069.00	ENSG00000159055	6.46	7.26	6.78	6.34	5.50	5.06
139231_at	FAM199X	− 1.20	− 2.29	− 4.94	0.00	0.85	− 4.26	Family with sequence similarity 199, X-linked	139231.00	ENSG00000123575	6.30	6.70	6.83	5.73	5.35	5.14
81502_at	HM13	− 1.20	− 2.30	− 2.64	0.04	0.85	− 4.40	Histocompatibility minor 13	81502.00	ENSG00000101294	6.54	6.81	7.20	6.04	6.24	4.66
81875_at	ISG20L2	− 1.20	− 2.30	− 2.49	0.05	0.85	− 4.42	Interferon stimulated exonuclease gene 20 like 2	81875.00	ENSG00000143319	6.63	6.70	7.71	6.03	6.46	4.94
10040_at	TOM1L1	− 1.22	− 2.32	− 2.98	0.02	0.85	− 4.37	Target of myb1 like 1 membrane trafficking protein	10040.00	ENSG00000141198	6.21	7.28	5.96	5.74	5.08	4.99
440138_at	ALG11	− 1.22	− 2.33	− 2.47	0.05	0.85	− 4.42	ALG11, alpha-1,2-mannosyltransferase	440138.00	ENSG00000253710	6.85	6.96	7.14	6.24	6.43	4.62
57669_at	EPB41L5	− 1.22	− 2.33	− 3.05	0.02	0.85	− 4.37	Erythrocyte membrane protein band 4.1 like 5	57669.00	ENSG00000115109	5.94	6.75	6.66	5.73	5.48	4.48
2120_at	ETV6	− 1.23	− 2.34	− 2.75	0.03	0.85	− 4.39	ETS variant 6	2120.00	ENSG00000139083	6.38	7.66	7.03	6.26	6.06	5.07
85461_at	TANC1	− 1.24	− 2.36	− 2.81	0.03	0.85	− 4.39	Tetratricopeptide repeat, ankyrin repeat and coiled-coil containing 1	85461.00	ENSG00000115183	6.36	7.37	6.29	5.86	5.76	4.69
127544_at	RNF19B	− 1.24	− 2.36	− 3.13	0.02	0.85	− 4.36	Ring finger protein 19B	127544.00	ENSG00000116514	5.77	6.76	6.27	5.32	5.44	4.33
8351_at	HIST1H3D	− 1.24	− 2.36	− 2.71	0.03	0.85	− 4.40	Histone cluster 1 H3 family member d	8351.00	ENSG00000197409	6.54	6.61	7.43	6.39	4.83	5.65
2762_at	GMDS	− 1.25	− 2.38	− 2.97	0.02	0.85	− 4.37	GDP-mannose 4,6-dehydratase	2762.00	ENSG00000112699	5.64	6.19	6.75	5.17	5.43	4.22
55298_at	RNF121	− 1.25	− 2.39	− 2.52	0.04	0.85	− 4.42	Ring finger protein 121	55298.00	ENSG00000137522	4.68	5.17	5.44	4.28	4.49	2.76
6809_at	STX3	− 1.26	− 2.39	− 3.23	0.02	0.85	− 4.35	Syntaxin 3	6809.00	ENSG00000166900	6.03	6.89	7.18	5.46	5.92	4.94
51514_at	DTL	− 1.26	− 2.39	− 3.24	0.02	0.85	− 4.35	Denticleless E3 ubiquitin protein ligase homolog	51514.00	ENSG00000143476	6.44	5.08	5.58	4.27	4.83	4.22
1903_at	S1PR3	− 1.26	− 2.40	− 3.11	0.02	0.85	− 4.36	Sphingosine-1-phosphate receptor 3	1903.00	ENSG00000213694	5.47	5.96	6.54	5.20	4.95	4.04
100288069_at	LOC100288069	− 1.27	− 2.40	− 3.58	0.01	0.85	− 4.33	Uncharacterized LOC100288069	100288069.00	NA	5.03	5.98	5.56	4.54	3.69	4.55
221079_at	ARL5B	− 1.27	− 2.41	− 2.86	0.03	0.85	− 4.38	ADP ribosylation factor like GTPase 5B	221079.00	ENSG00000165997	6.61	8.07	7.89	6.46	6.53	5.78
72_at	ACTG2	− 1.27	− 2.41	− 3.35	0.01	0.85	− 4.35	Actin, gamma 2, smooth muscle, enteric	72.00	ENSG00000163017	3.76	4.44	3.10	2.36	2.87	2.25
23636_at	NUP62	− 1.27	− 2.41	− 2.97	0.02	0.85	− 4.37	Nucleoporin 62	23636.00	ENSG00000213024	5.99	6.14	7.01	5.40	5.58	4.35
57154_at	SMURF1	− 1.28	− 2.43	− 2.97	0.02	0.85	− 4.37	SMAD specific E3 ubiquitin protein ligase 1	57154.00	ENSG00000198742, ENSG00000284126	5.85	6.76	5.71	5.32	5.08	4.08
9055_at	PRC1	− 1.28	− 2.43	− 4.05	0.01	0.85	− 4.30	Protein regulator of cytokinesis 1	9055.00	ENSG00000198901	7.49	7.99	7.11	6.27	6.64	5.83
201475_at	RAB12	− 1.28	− 2.43	− 2.88	0.03	0.85	− 4.38	RAB12, member RAS oncogene family	201475.00	ENSG00000206418	7.79	8.27	7.74	6.98	7.29	5.68
11123_at	RCAN3	− 1.30	− 2.46	− 2.97	0.02	0.85	− 4.37	RCAN family member 3	11123.00	ENSG00000117602	5.67	7.23	6.70	5.67	5.10	4.94
10276_at	NET1	− 1.30	− 2.46	− 3.19	0.02	0.85	− 4.36	Neuroepithelial cell transforming 1	10276.00	ENSG00000173848	6.53	7.72	6.78	5.85	6.14	5.14
79065_at	ATG9A	− 1.30	− 2.47	− 3.05	0.02	0.85	− 4.37	Autophagy related 9A	79065.00	ENSG00000198925	6.34	7.11	7.46	5.94	6.12	4.93
890_at	CCNA2	− 1.31	− 2.47	− 2.50	0.05	0.85	− 4.42	cyclin A2	890.00	ENSG00000145386	6.91	6.72	5.52	5.40	5.63	4.20
100506658_at	OCLN	− 1.31	− 2.48	− 3.25	0.02	0.85	− 4.35	occludin	100506658.00	ENSG00000197822, ENSG00000273814	3.76	5.05	5.21	3.44	3.42	3.24
5209_at	PFKFB3	− 1.31	− 2.49	− 2.46	0.05	0.85	− 4.42	6-Phosphofructo-2-kinase/fructose-2,6-biphosphatase 3	5209.00	ENSG00000170525	4.95	5.48	6.21	4.44	5.01	3.25
51361_at	HOOK1	− 1.31	− 2.49	− 2.65	0.04	0.85	− 4.40	Hook microtubule tethering protein 1	51361.00	ENSG00000134709	3.25	4.78	5.06	2.77	3.04	3.34
701_at	BUB1B	− 1.32	− 2.50	− 5.79	0.00	0.85	− 4.24	BUB1 mitotic checkpoint serine/threonine kinase B	701.00	ENSG00000156970	6.46	6.40	5.91	5.01	4.70	5.10
85406_at	DNAJC14	− 1.33	− 2.52	− 3.14	0.02	0.85	− 4.36	DnaJ heat shock protein family (Hsp40) member C14	85406.00	ENSG00000135392	5.91	6.24	6.27	5.19	5.38	3.85
6907_at	TBL1X	− 1.34	− 2.53	− 3.73	0.01	0.85	− 4.32	Transducin beta like 1 X-linked	6907.00	ENSG00000101849	4.83	5.79	4.97	4.30	3.90	3.38
3728_at	JUP	− 1.35	− 2.54	− 2.69	0.04	0.85	− 4.40	Junction plakoglobin	3728.00	ENSG00000173801	5.39	7.24	6.88	4.82	5.41	5.25
7153_at	TOP2A	− 1.35	− 2.55	− 2.47	0.05	0.85	− 4.42	DNA topoisomerase II alpha	7153.00	ENSG00000131747	8.13	6.98	7.44	6.45	6.96	5.08
56888_at	KCMF1	− 1.35	− 2.56	− 2.57	0.04	0.85	− 4.41	Potassium channel modulatory factor 1	56888.00	ENSG00000176407	7.45	8.10	8.39	7.40	6.94	5.54
112616_at	CMTM7	− 1.35	− 2.56	− 2.58	0.04	0.85	− 4.41	CKLF like MARVEL transmembrane domain containing 7	112616.00	ENSG00000153551	5.31	7.09	6.28	4.46	5.58	4.57
4318_at	MMP9	− 1.36	− 2.57	− 2.86	0.03	0.85	− 4.38	Matrix metallopeptidase 9	4318.00	ENSG00000100985	5.69	6.61	7.44	5.56	5.32	4.77
57535_at	KIAA1324	− 1.36	− 2.57	− 3.51	0.01	0.85	− 4.33	KIAA1324	57535.00	ENSG00000116299	5.17	6.65	5.85	4.34	4.75	4.49
57162_at	PELI1	− 1.38	− 2.60	− 2.82	0.03	0.85	− 4.39	Pellino E3 ubiquitin protein ligase 1	57162.00	ENSG00000197329	5.32	7.19	6.58	5.08	5.22	4.64
9654_at	TTLL4	− 1.38	− 2.60	− 2.48	0.05	0.85	− 4.42	Tubulin tyrosine ligase like 4	9654.00	ENSG00000135912	6.28	7.67	8.29	6.19	6.45	5.46
493856_at	CISD2	− 1.39	− 2.61	− 4.51	0.00	0.85	− 4.28	CDGSH iron sulfur domain 2	493856.00	ENSG00000145354	6.53	7.52	7.37	6.00	5.65	5.61
1857_at	DVL3	− 1.39	− 2.62	− 2.90	0.03	0.85	− 4.38	Dishevelled segment polarity protein 3	1857.00	ENSG00000161202	7.03	8.07	7.79	6.77	6.66	5.30
102723739_at	LOC102723739	− 1.39	− 2.63	− 2.66	0.04	0.85	− 4.40	Uncharacterized LOC102723739	102723739.00	NA	3.14	4.09	5.29	2.79	2.80	2.75
91452_at	ACBD5	− 1.40	− 2.63	− 2.44	0.05	0.85	− 4.42	Acyl-CoA binding domain containing 5	91452.00	ENSG00000107897	5.59	7.02	5.87	5.24	5.35	3.71
79622_at	SNRNP25	− 1.41	− 2.66	− 3.19	0.02	0.85	− 4.36	Small nuclear ribonucleoprotein U11/U12 subunit 25	79622.00	ENSG00000161981	5.44	6.73	6.20	4.54	5.39	4.20
200634_at	KRTCAP3	− 1.41	− 2.66	− 2.79	0.03	0.85	− 4.39	Keratinocyte associated protein 3	200634.00	ENSG00000157992	5.96	7.90	6.93	5.59	5.84	5.12
6533_at	SLC6A6	− 1.41	− 2.66	− 2.61	0.04	0.85	− 4.41	Solute carrier family 6 member 6	6533.00	ENSG00000131389	6.44	7.15	8.29	5.87	6.50	5.28
3419_at	IDH3A	− 1.41	− 2.67	− 2.56	0.04	0.85	− 4.41	Isocitrate dehydrogenase 3 (NAD(+)) alpha	3419.00	ENSG00000166411	6.50	7.78	6.68	6.24	5.95	4.54
85236_at	HIST1H2BK	− 1.42	− 2.67	− 2.84	0.03	0.85	− 4.39	Histone cluster 1 H2B family member k	85236.00	ENSG00000197903	8.26	7.61	8.77	7.17	7.39	5.83
4133_at	MAP2	− 1.42	− 2.68	− 2.67	0.04	0.85	− 4.40	Microtubule associated protein 2	4133.00	ENSG00000078018	5.20	7.30	5.83	4.86	4.76	4.45
10809_at	STARD10	− 1.42	− 2.68	− 2.82	0.03	0.85	− 4.39	StAR related lipid transfer domain containing 10	10809.00	ENSG00000214530	5.74	7.20	6.04	4.20	4.94	5.57
440278_at	CATSPER2P1	− 1.43	− 2.70	− 3.43	0.01	0.85	− 4.34	Cation channel sperm associated 2 pseudogene 1	440278.00	ENSG00000205771	4.41	5.58	4.78	4.17	3.16	3.15
8985_at	PLOD3	− 1.46	− 2.74	− 3.09	0.02	0.85	− 4.36	Procollagen-lysine,2-oxoglutarate 5-dioxygenase 3	8985.00	ENSG00000106397	7.17	8.09	8.06	6.92	6.63	5.41
7978_at	MTERF1	− 1.46	− 2.74	− 3.13	0.02	0.85	− 4.36	Mitochondrial transcription termination factor 1	7978.00	ENSG00000127989	5.75	5.78	5.99	4.98	3.31	4.87
146223_at	CMTM4	− 1.47	− 2.78	− 4.22	0.01	0.85	− 4.29	CKLF like MARVEL transmembrane domain containing 4	146223.00	ENSG00000183723	6.79	7.29	6.90	5.74	6.00	4.82
64426_at	SUDS3	− 1.48	− 2.78	− 3.91	0.01	0.85	− 4.31	SDS3 homolog, SIN3A corepressor complex component	64426.00	ENSG00000111707	5.36	5.85	6.04	4.52	4.77	3.53
57530_at	CGN	− 1.48	− 2.79	− 2.47	0.05	0.85	− 4.42	Cingulin	57530.00	ENSG00000143375	3.80	5.94	5.36	2.96	4.09	3.61
6271_at	S100A1	− 1.48	− 2.79	− 2.43	0.05	0.85	− 4.42	S100 calcium binding protein A1	6271.00	ENSG00000160678	4.99	7.06	7.09	4.48	4.99	5.24
375035_at	SFT2D2	− 1.49	− 2.81	− 2.72	0.03	0.85	− 4.40	SFT2 domain containing 2	375035.00	ENSG00000213064	8.02	8.63	9.94	7.04	8.03	7.05
158586_at	ZXDB	− 1.50	− 2.82	− 2.98	0.02	0.85	− 4.37	Zinc finger, X-linked, duplicated B	158586.00	ENSG00000198455	6.61	7.35	6.90	6.50	5.24	4.62
9368_at	SLC9A3R1	− 1.50	− 2.82	− 3.06	0.02	0.85	− 4.37	SLC9A3 regulator 1	9368.00	ENSG00000109062	6.51	8.26	7.59	5.91	6.44	5.52
6398_at	SECTM1	− 1.50	− 2.83	− 2.51	0.04	0.85	− 4.42	Secreted and transmembrane 1	6398.00	ENSG00000141574	5.80	6.88	8.25	5.56	5.25	5.61
1717_at	DHCR7	− 1.51	− 2.84	− 2.91	0.03	0.85	− 4.38	7-Dehydrocholesterol reductase	1717.00	ENSG00000172893	6.16	8.01	7.10	6.15	5.50	5.10
7097_at	TLR2	− 1.51	− 2.84	− 3.05	0.02	0.85	− 4.37	Toll like receptor 2	7097.00	ENSG00000137462	5.32	6.65	6.31	3.74	5.13	4.89
94039_at	ZNF101	− 1.53	− 2.90	− 2.44	0.05	0.85	− 4.42	Zinc finger protein 101	94039.00	ENSG00000181896	4.28	6.82	5.07	3.94	3.99	3.64
22974_at	TPX2	− 1.54	− 2.91	− 3.85	0.01	0.85	− 4.31	TPX2, microtubule nucleation factor	22974.00	ENSG00000088325	6.77	6.86	6.44	5.59	5.59	4.27
1838_at	DTNB	− 1.55	− 2.92	− 3.70	0.01	0.85	− 4.32	Dystrobrevin beta	1838.00	ENSG00000138101	4.93	5.76	6.59	4.31	4.17	4.17
51176_at	LEF1	− 1.55	− 2.92	− 3.59	0.01	0.85	− 4.33	Lymphoid enhancer binding factor 1	51176.00	ENSG00000138795	4.60	4.50	5.54	4.01	2.71	3.28
10207_at	PATJ	− 1.55	− 2.94	− 3.54	0.01	0.85	− 4.33	PATJ, crumbs cell polarity complex component	10207.00	ENSG00000132849	5.44	7.03	6.29	5.14	4.59	4.38
29028_at	ATAD2	− 1.56	− 2.95	− 3.11	0.02	0.85	− 4.36	ATPase family, AAA domain containing 2	29028.00	ENSG00000156802	6.59	6.84	6.37	5.91	5.26	3.94
4301_at	AFDN	− 1.58	− 2.99	− 3.08	0.02	0.85	− 4.37	Afadin, adherens junction formation factor	4301.00	ENSG00000130396	7.28	8.54	7.96	6.92	6.74	5.38
3838_at	KPNA2	− 1.58	− 2.99	− 2.62	0.04	0.85	− 4.41	Karyopherin subunit alpha 2	3838.00	ENSG00000182481	7.53	7.88	7.85	7.19	6.54	4.78
79837_at	PIP4K2C	− 1.58	− 2.99	− 2.43	0.05	0.85	− 4.42	Phosphatidylinositol-5-phosphate 4-kinase type 2 gamma	79837.00	ENSG00000166908	5.02	7.63	6.73	4.54	5.18	4.91
3691_at	ITGB4	− 1.59	− 3.01	− 3.59	0.01	0.85	− 4.33	Integrin subunit beta 4	3691.00	ENSG00000132470	4.89	6.44	6.02	3.86	4.64	4.08
8342_at	HIST1H2BM	− 1.59	− 3.02	− 7.74	0.00	0.85	− 4.21	Histone cluster 1 H2B family member m	8342.00	ENSG00000273703	6.08	6.15	6.49	4.88	4.55	4.50
1317_at	SLC31A1	− 1.60	− 3.03	− 2.62	0.04	0.85	− 4.41	Solute carrier family 31 member 1	1317.00	ENSG00000136868	7.61	8.97	8.40	7.02	7.62	5.54
360019_at	KRT18P10	− 1.61	− 3.05	− 3.19	0.02	0.85	− 4.36	Keratin 18 pseudogene 10	360019.00	NA	3.49	5.09	3.33	2.02	2.32	2.73
388564_at	TMEM238	− 1.62	− 3.07	− 2.91	0.03	0.85	− 4.38	Transmembrane protein 238	388564.00	ENSG00000233493	5.09	6.52	7.12	5.18	4.36	4.34
4316_at	MMP7	− 1.62	− 3.07	− 2.79	0.03	0.85	− 4.39	Matrix metallopeptidase 7	4316.00	ENSG00000137673	6.90	8.39	6.73	6.19	6.15	4.82
27350_at	APOBEC3C	− 1.62	− 3.08	− 3.59	0.01	0.85	− 4.33	Apolipoprotein B mRNA editing enzyme catalytic subunit 3C	27350.00	ENSG00000244509	6.78	6.83	7.46	6.07	5.66	4.47
26578_at	OSTF1	− 1.62	− 3.08	− 2.59	0.04	0.85	− 4.41	Osteoclast stimulating factor 1	26578.00	ENSG00000134996	6.81	7.32	7.15	6.36	6.06	4.00
8424_at	BBOX1	− 1.62	− 3.08	− 2.43	0.05	0.85	− 4.43	Gamma-Butyrobetaine hydroxylase 1	8424.00	ENSG00000129151	3.19	4.73	5.98	2.95	3.10	2.97
56990_at	CDC42SE2	− 1.63	− 3.10	− 2.83	0.03	0.85	− 4.39	CDC42 small effector 2	56990.00	ENSG00000158985	6.36	6.43	6.68	5.77	5.28	3.52
27095_at	TRAPPC3	− 1.64	− 3.11	− 2.68	0.04	0.85	− 4.40	Trafficking protein particle complex 3	27095.00	ENSG00000054116	7.43	8.62	9.34	7.39	7.21	5.87
1672_at	DEFB1	− 1.64	− 3.12	− 2.76	0.03	0.85	− 4.39	Defensin beta 1	1672.00	ENSG00000164825	6.43	8.53	7.29	5.20	6.46	5.67
7045_at	TGFBI	− 1.65	− 3.13	− 2.51	0.04	0.85	− 4.42	Transforming growth factor beta induced	7045.00	ENSG00000120708	8.86	7.38	7.93	6.92	7.22	5.10
105374310_at	LOC105374310	− 1.65	− 3.15	− 3.11	0.02	0.85	− 4.36	Uncharacterized LOC105374310	105374310.00	NA	4.90	6.39	6.91	4.27	4.80	4.16
1836_at	SLC26A2	− 1.67	− 3.17	− 2.90	0.03	0.85	− 4.38	Solute carrier family 26 member 2	1836.00	ENSG00000155850	6.09	7.64	6.78	5.93	5.41	4.17
196_at	AHR	− 1.68	− 3.21	− 3.62	0.01	0.85	− 4.33	Aryl hydrocarbon receptor	196.00	ENSG00000106546	8.48	9.96	8.80	7.66	7.75	6.77
4233_at	MET	− 1.70	− 3.24	− 2.82	0.03	0.85	− 4.39	MET proto-oncogene, receptor tyrosine kinase	4233.00	ENSG00000105976	6.25	6.90	5.63	5.72	4.39	3.58
10652_at	YKT6	− 1.70	− 3.24	− 3.35	0.01	0.85	− 4.34	YKT6 v-SNARE homolog	10652.00	ENSG00000106636	6.29	7.03	7.09	5.77	5.51	4.03
5768_at	QSOX1	− 1.70	− 3.25	− 2.53	0.04	0.85	− 4.41	Quiescin sulfhydryl oxidase 1	5768.00	ENSG00000116260	5.27	7.66	7.06	5.27	5.40	4.22
93474_at	ZNF670	− 1.72	− 3.30	− 5.97	0.00	0.85	− 4.24	Zinc finger protein 670	93474.00	ENSG00000277462	4.16	4.70	4.86	3.29	2.65	2.61
7020_at	TFAP2A	− 1.74	− 3.34	− 2.58	0.04	0.85	− 4.41	Transcription factor AP-2 alpha	7020.00	ENSG00000137203	5.64	6.83	8.41	5.11	5.46	5.09
11221_at	DUSP10	− 1.74	− 3.34	− 3.52	0.01	0.85	− 4.33	Dual specificity phosphatase 10	11221.00	ENSG00000143507	7.27	7.36	8.15	6.18	6.53	4.85
56900_at	TMEM167B	− 1.75	− 3.36	− 2.51	0.04	0.85	− 4.42	Transmembrane protein 167B	56900.00	ENSG00000215717	6.05	7.34	7.33	5.78	5.98	3.71
5557_at	PRIM1	− 1.77	− 3.42	− 3.72	0.01	0.85	− 4.32	DNA primase subunit 1	5557.00	ENSG00000198056	5.69	6.34	5.89	5.24	3.85	3.52
5795_at	PTPRJ	− 1.78	− 3.45	− 2.69	0.04	0.85	− 4.40	Protein tyrosine phosphatase, receptor type J	5795.00	ENSG00000149177	5.39	8.03	6.45	4.69	5.31	4.51
79022_at	TMEM106C	− 1.79	− 3.46	− 2.53	0.04	0.85	− 4.41	Transmembrane protein 106C	79022.00	ENSG00000134291	5.41	7.66	6.77	5.50	5.25	3.73
481_at	ATP1B1	− 1.80	− 3.47	− 2.47	0.05	0.85	− 4.42	ATPase Na+/K+ transporting subunit beta 1	481.00	ENSG00000143153	7.64	10.31	8.18	6.69	7.58	6.48
105376171_at	LOC105376171	− 1.81	− 3.51	− 3.93	0.01	0.85	− 4.31	Uncharacterized LOC105376171	105376171.00	ENSG00000231521	5.55	6.06	6.77	5.09	4.15	3.71
283987_at	HID1	− 1.84	− 3.59	− 3.15	0.02	0.85	− 4.36	HID1 domain containing	283987.00	ENSG00000167861	5.14	7.50	6.41	4.34	4.84	4.34
1942_at	EFNA1	− 1.85	− 3.59	− 3.36	0.01	0.85	− 4.34	Ephrin A1	1942.00	ENSG00000169242	3.73	5.03	5.96	2.89	3.02	3.27
84844_at	PHF5A	− 1.85	− 3.60	− 2.79	0.03	0.85	− 4.39	PHD finger protein 5A	84844.00	ENSG00000100410	4.91	6.55	7.50	4.70	3.97	4.75
10653_at	SPINT2	− 1.86	− 3.63	− 2.56	0.04	0.85	− 4.41	Serine peptidase inhibitor, Kunitz type 2	10653.00	ENSG00000167642	5.07	7.84	6.95	4.08	5.09	5.10
100313770_at	MIR548K	− 1.87	− 3.66	− 2.88	0.03	0.85	− 4.38	MicroRNA 548k	100313770.00	ENSG00000221333	3.67	4.79	6.36	3.00	3.16	3.05
83481_at	EPPK1	− 1.90	− 3.73	− 2.96	0.02	0.85	− 4.38	Epiplakin 1	83481.00	ENSG00000261150	3.92	5.27	6.60	3.38	3.37	3.34
55971_at	BAIAP2L1	− 1.90	− 3.74	− 2.68	0.04	0.85	− 4.40	BAI1 associated protein 2 like 1	55971.00	ENSG00000006453	5.12	7.77	6.23	4.14	5.25	4.03
3675_at	ITGA3	− 1.91	− 3.75	− 2.69	0.03	0.85	− 4.40	Integrin subunit alpha 3	3675.00	ENSG00000005884	6.84	7.81	7.05	6.01	6.25	3.72
1545_at	CYP1B1	− 1.95	− 3.86	− 2.71	0.03	0.85	− 4.40	Cytochrome P450 family 1 subfamily B member 1	1545.00	ENSG00000138061	7.76	8.41	9.92	7.13	7.54	5.58
57153_at	SLC44A2	− 1.96	− 3.88	− 2.62	0.04	0.85	− 4.41	Solute carrier family 44 member 2	57153.00	ENSG00000129353	6.65	8.62	8.63	6.29	6.93	4.82
11009_at	IL24	− 1.96	− 3.90	− 2.95	0.02	0.85	− 4.38	Interleukin 24	11009.00	ENSG00000162892	6.61	7.42	7.13	4.36	6.63	4.28
6272_at	SORT1	− 1.99	− 3.97	− 2.78	0.03	0.85	− 4.39	Sortilin 1	6272.00	ENSG00000134243	5.51	7.42	7.93	5.07	5.70	4.12
6692_at	SPINT1	− 2.00	− 4.01	− 3.35	0.01	0.85	− 4.35	Serine peptidase inhibitor, Kunitz type 1	6692.00	ENSG00000166145	5.61	8.07	6.52	4.84	4.67	4.68
129642_at	MBOAT2	− 2.01	− 4.03	− 3.80	0.01	0.85	− 4.32	Membrane bound *O*-acyltransferase domain containing 2	129642.00	ENSG00000143797	6.45	7.14	6.80	5.33	5.43	3.59
1829_at	DSG2	− 2.01	− 4.04	− 3.59	0.01	0.85	− 4.33	Desmoglein 2	1829.00	ENSG00000046604	6.13	8.00	6.21	4.67	5.29	4.34
92421_at	CHMP4C	− 2.07	− 4.19	− 2.76	0.03	0.85	− 4.39	Charged multivesicular body protein 4C	92421.00	ENSG00000164695	4.82	7.95	6.36	4.17	4.22	4.54
219970_at	GLYATL2	− 2.07	− 4.19	− 5.74	0.00	0.85	− 4.24	Glycine-*N*-acyltransferase like 2	219970.00	ENSG00000156689	6.74	6.55	6.57	5.30	4.44	3.92
1366_at	CLDN7	− 2.10	− 4.30	− 3.20	0.02	0.85	− 4.36	Claudin 7	1366.00	ENSG00000181885	5.76	8.28	7.54	4.96	5.60	4.71
55041_at	PLEKHB2	− 2.12	− 4.34	− 2.52	0.04	0.85	− 4.42	Pleckstrin homology domain containing B2	55041.00	ENSG00000115762	5.98	7.53	7.36	5.85	5.63	3.04
337875_at	HIST2H2BA	− 2.16	− 4.47	− 3.17	0.02	0.85	− 4.36	Histone cluster 2 H2B family member a (pseudogene)	337875.00	NA	5.74	6.89	7.36	5.17	5.17	3.17
6768_at	ST14	− 2.18	− 4.53	− 2.65	0.04	0.85	− 4.40	Suppression of tumorigenicity 14	6768.00	ENSG00000149418	4.10	7.39	6.61	3.59	4.00	3.98
8140_at	SLC7A5	− 2.20	− 4.58	− 2.99	0.02	0.85	− 4.37	Solute carrier family 7 member 5	8140.00	ENSG00000103257	6.75	9.18	8.08	5.84	6.74	4.84
1824_at	DSC2	− 2.20	− 4.61	− 3.51	0.01	0.85	− 4.33	Desmocollin 2	1824.00	ENSG00000134755	7.20	9.00	8.68	6.43	6.77	5.07
200058_at	FLJ23867	− 2.20	− 4.61	− 3.11	0.02	0.85	− 4.36	Uncharacterized protein FLJ23867	200058.00	NA	6.24	9.21	8.00	5.63	5.62	5.59
51330_at	TNFRSF12A	− 2.28	− 4.86	− 2.71	0.03	0.85	− 4.40	TNF receptor superfamily member 12A	51330.00	ENSG00000006327	6.98	8.53	10.21	5.90	7.16	5.82
2517_at	FUCA1	− 2.31	− 4.97	− 2.64	0.04	0.85	− 4.40	alpha-L-Fucosidase 1	2517.00	ENSG00000179163	5.26	7.76	8.62	5.33	5.19	4.17
1832_at	DSP	− 2.34	− 5.06	− 6.25	0.00	0.85	− 4.23	Desmoplakin	1832.00	ENSG00000096696	7.04	8.21	8.17	5.74	5.56	5.11
1500_at	CTNND1	− 2.38	− 5.22	− 2.78	0.03	0.85	− 4.39	Catenin delta 1	1500.00	ENSG00000198561	6.60	9.08	8.82	5.74	7.01	4.60
55959_at	SULF2	− 2.39	− 5.24	− 3.21	0.02	0.85	− 4.36	Sulfatase 2	55959.00	ENSG00000196562	6.43	8.46	6.99	4.71	6.15	3.84
102723517_at	LOC102723517	− 2.42	− 5.34	− 2.87	0.03	0.85	− 4.38	Uncharacterized LOC102723517	102723517.00	ENSG00000234899	7.56	9.83	10.72	6.94	7.68	6.25
79679_at	VTCN1	− 2.42	− 5.36	− 2.75	0.03	0.85	− 4.39	V-set domain containing T-cell activation inhibitor 1	79679.00	ENSG00000134258	4.11	6.81	7.68	3.76	3.66	3.92
50848_at	F11R	− 2.45	− 5.45	− 2.56	0.04	0.85	− 4.41	F11 receptor	50848.00	ENSG00000158769	5.11	8.90	7.36	4.60	5.42	4.00
4071_at	TM4SF1	− 2.46	− 5.50	− 2.70	0.03	0.85	− 4.40	Transmembrane 4 L six family member 1	4071.00	ENSG00000169908	5.87	9.24	7.98	5.71	5.82	4.19
999_at	CDH1	− 2.46	− 5.50	− 3.13	0.02	0.85	− 4.36	Cadherin 1	999.00	ENSG00000039068	6.21	9.33	6.90	4.97	5.32	4.78
2568_at	GABRP	− 2.51	− 5.70	− 2.92	0.03	0.85	− 4.38	gamma-Aminobutyric acid type A receptor pi subunit	2568.00	ENSG00000094755	5.83	8.82	8.48	4.59	6.09	4.90
54845_at	ESRP1	− 2.56	− 5.91	− 2.58	0.04	0.85	− 4.41	Epithelial splicing regulatory protein 1	54845.00	ENSG00000104413	3.97	8.06	6.65	3.27	4.06	3.66
3655_at	ITGA6	− 2.58	− 5.97	− 2.73	0.03	0.85	− 4.40	Integrin subunit alpha 6	3655.00	ENSG00000091409	5.87	9.56	8.76	4.93	5.84	5.69
3854_at	KRT6B	− 2.61	− 6.11	− 2.62	0.04	0.85	− 4.41	Keratin 6B	3854.00	ENSG00000185479	9.02	9.60	9.42	8.23	7.64	4.34
3694_at	ITGB6	− 2.63	− 6.18	− 5.73	0.00	0.85	− 4.24	Integrin subunit beta 6	3694.00	ENSG00000115221	7.21	8.24	7.04	4.28	5.59	4.73
27075_at	TSPAN13	− 2.75	− 6.73	− 2.70	0.03	0.85	− 4.40	Tetraspanin 13	27075.00	ENSG00000106537	5.26	9.38	8.10	4.39	4.75	5.35
654319_at	SNORA5A	− 2.76	− 6.79	− 3.19	0.02	0.85	− 4.36	Small nucleolar RNA, H/ACA box 5A	654319.00	ENSG00000206838	3.56	6.90	6.28	3.32	2.69	2.45
8842_at	PROM1	− 2.76	− 6.79	− 2.56	0.04	0.85	− 4.41	Prominin 1	8842.00	ENSG00000007062	5.98	9.88	7.29	4.82	6.17	3.86
102723505_at	LINC02095	− 2.86	− 7.25	− 2.67	0.04	0.85	− 4.40	Long intergenic non-protein coding RNA 2095	102723505.00	ENSG00000228639	5.17	8.97	8.94	4.90	5.43	4.17
114569_at	MAL2	− 3.05	− 8.28	− 3.35	0.01	0.85	− 4.34	mal, T-cell differentiation protein 2 (gene/pseudogene)	114569.00	ENSG00000147676	7.95	10.23	9.52	6.32	7.65	4.58
938_at	CD24P4	− 3.29	− 9.81	− 3.13	0.02	0.85	− 4.36	CD24 molecule pseudogene 4	938.00	NA	6.48	7.89	7.12	3.49	6.16	1.96
105376425_at	LOC105376425	− 3.46	-11.03	− 3.40	0.01	0.85	− 4.34	Uncharacterized LOC105376425	105376425.00	NA	6.44	9.61	6.90	5.20	4.68	2.68
8364_at	HIST1H4C	− 3.63	-12.39	− 3.25	0.02	0.85	− 4.35	Histone cluster 1 H4 family member c	8364.00	ENSG00000197061	6.78	5.03	9.38	4.50	2.75	3.05
260436_at	FDCSP	− 4.42	-21.46	− 2.81	0.03	0.85	− 4.39	Follicular dendritic cell secreted protein	260436.00	ENSG00000181617	8.08	11.32	11.19	4.13	9.03	4.16
6279_at	S100A8	− 4.79	-27.66	− 3.93	0.01	0.85	− ρ4.31	S100 calcium binding protein A8	6279.00	ENSG00000143546	5.66	9.82	10.42	3.96	3.56	4.01

BT133, BT134 and BT138 corresponds to three different TNBC patients

### Discussion

Although one study did examine the impact of cryopreservation on gametes and embryos, this study appears to be the first to examine phenotypic changes caused by freeze/thawing living cells isolated from cancer patients [7].

Although molecular oncology has opened new avenues to classifying human cancers from a molecular standpoint, a number of issues associated with heterogeneous genomic platforms limit their ability to identifying signatures capable of predicting biological behavior and/or identifying new molecular targets for more effective and less toxic therapeutic interventions [8]. We identified a novel issue using data meta-analysis based on profiling of fresh and frozen tumor samples; our findings suggest that it will be difficult to identify robust signatures transposable to patients if freeze/thawed dissociated cancer tissues are used.

We strongly discourage the use of frozen dissociated tumor sample for accurate cell population characterization, as thawing creates a disequilibrium in the proportion of immune and tumor cell subpopulations. In addition, because the transcriptomic signatures of mammospheres derived from frozen/thawed samples are not representative of those of initial CSCs, this study rules out their use for identification of new prognostic and/or therapeutic targets. We strongly suggest that future studies involving dissociated tumor cells should be conducted using fresh tumors only; in parallel, tumor biobanks should develop validate methods of freezing living cells isolated from resected tumors that preserves tumor heterogeneity. In this way, we may be able to generate robust scoring systems for prognostic, predictive, and therapeutic markers. Such a system is an unmet clinical need with respect to patients with TNBC.

## Limitations

Although, the freeze–thaw shock seems mainly to affect immune cells, a more detailed analysis would be required to further investigate whether some minor immune subsets could be affected by this stress and thereby, could also alter the conclusions drawn from analyses performed using freeze/thaw living cells.

## Additional files

**Additional file 1: Figure S1.** Flowchart showing the TNBC dissociation and spheroid protocols.

**Additional file 2: Table S1.** Gene ontology enrichment associated with the freeze/thaw process in TNBCs.
