# Supplementary material for: Tumor analysis: freeze–thawing cycle of triple-negative breast cancer cells alters tumor CD24/CD44 profiles and the percentage of tumor-infiltrating immune cells
Source: BMC Res Notes. 2018 Jun 20;11:401. doi: 10.1186/s13104-018-3504-5 (PMC6011598; doi:10.1186/s13104-018-3504-5)
Supplement: Supplementary file 1 — Additional file 1: Figure S1. Flowchart showing the TNBC dissociation and spheroid protocols. [file 13104_2018_3504_MOESM1_ESM.pptx]

## Slide 1
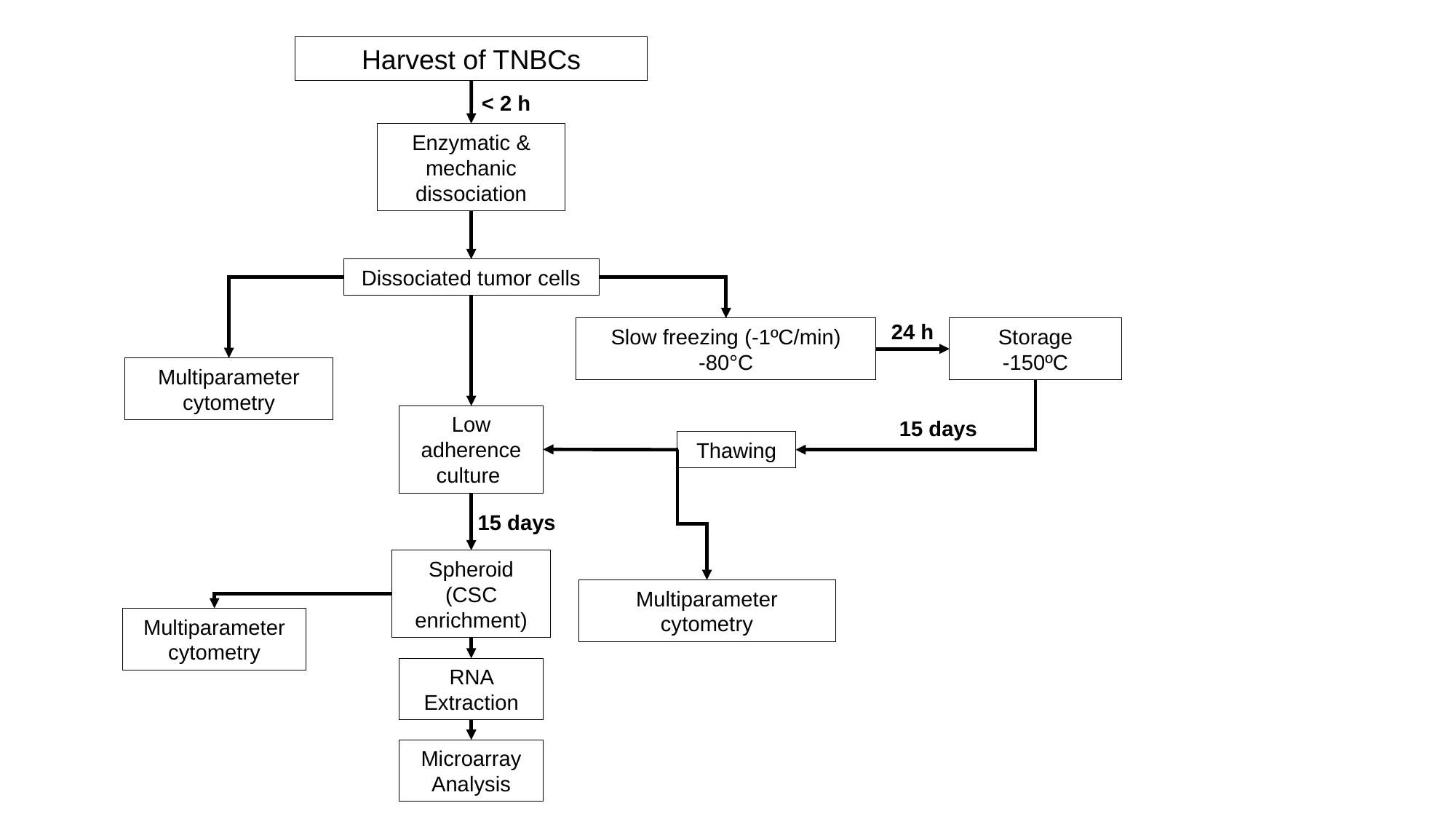

Harvest of TNBCs
< 2 h
Enzymatic & mechanic dissociation
Dissociated tumor cells
24 h
Slow freezing (-1ºC/min)
-80°C
Storage
-150ºC
Multiparameter cytometry
Low adherence culture
15 days
Thawing
15 days
Spheroid (CSC enrichment)
Multiparameter cytometry
Multiparameter cytometry
RNA Extraction
Microarray
Analysis
