# Supplementary material for: Tumor analysis: freeze–thawing cycle of triple-negative breast cancer cells alters tumor CD24/CD44 profiles and the percentage of tumor-infiltrating immune cells
Source: BMC Res Notes. 2018 Jun 20;11:401. doi: 10.1186/s13104-018-3504-5 (PMC6011598; doi:10.1186/s13104-018-3504-5)
Supplement: Supplementary file 2 — Additional file 2: Table S1. Gene ontology enrichment associated with the freeze/thaw process in TNBCs. [file 13104_2018_3504_MOESM2_ESM.docx]

| **#term** | **term_desc** | **term_size** | | **term_size_in_genome** | | **list1_positives** | | **list1_negatives** | | **list1_percentage** | | **list2_positives** | | **list2_negatives** | | **list2_percentage** | | **list1_positive_ids** | | **odds_ratio_log** | | **pvalue** | | **adj_pvalue** |
| --- | --- | --- | --- | --- | --- | --- | --- | --- | --- | --- | --- | --- | --- | --- | --- | --- | --- | --- | --- | --- | --- | --- | --- | --- |
| GO:0045216 | cell-cell junction organization | 464 | 508 | | 13 | | 211 | | 5.8 | | 451 | | 206873 | | 0.22 | | DLG1,SNAI1,EPB41L5,PLEKHA7,CDH1,F11R,OCLN,PTPRJ,CGN,DSP,JUP,NF2,CTNND1 | | 3.34E+00 | | 7.74E-15 | | 6.71E-11 | |
| GO:0007398 | ectoderm development | 44 | 51 | | 6 | | 218 | | 2.68 | | 38 | | 207286 | | 0.02 | | EPB41L5,SMURF1,JUP,NF2,ITGA6,KRT6B | | 5.01E+00 | | 1.01E-11 | | 4.37E-08 | |
| GO:0086069 | bundle of His cell to Purkinje myocyte communication | 12 | 16 | | 4 | | 220 | | 1.79 | | 8 | | 207316 | | 0 | | DSG2,DSC2,DSP,JUP | | 6.16E+00 | | 6.49E-10 | | 1.88E-06 | |
| GO:0070830 | tight junction assembly | 100 | 111 | | 6 | | 218 | | 2.68 | | 94 | | 207230 | | 0.05 | | DLG1,SNAI1,CDH1,F11R,OCLN,CGN | | 4.11E+00 | | 1.62E-09 | | 2.81E-06 | |
| GO:0007043 | cell-cell junction assembly | 178 | 191 | | 7 | | 217 | | 3.12 | | 171 | | 207153 | | 0.08 | | DLG1,SNAI1,CDH1,F11R,OCLN,CGN,JUP | | 3.67E+00 | | 1.32E-09 | | 2.81E-06 | |
| GO:0001707 | mesoderm formation | 105 | 113 | | 6 | | 218 | | 2.68 | | 99 | | 207225 | | 0.05 | | SNAI1,EPB41L5,NF2,ITGB4,ITGA3,LEF1 | | 4.05E+00 | | 2.17E-09 | | 3.14E-06 | |
| GO:0048332 | mesoderm morphogenesis | 109 | 117 | | 6 | | 218 | | 2.68 | | 103 | | 207221 | | 0.05 | | SNAI1,EPB41L5,NF2,ITGB4,ITGA3,LEF1 | | 4.01E+00 | | 2.73E-09 | | 3.38E-06 | |
| GO:0043297 | apical junction assembly | 118 | 129 | | 6 | | 218 | | 2.68 | | 112 | | 207212 | | 0.05 | | DLG1,SNAI1,CDH1,F11R,OCLN,CGN | | 3.93E+00 | | 4.40E-09 | | 4.77E-06 | |
| GO:0007498 | mesoderm development | 230 | 238 | | 7 | | 217 | | 3.12 | | 223 | | 207101 | | 0.11 | | SECTM1,SNAI1,EPB41L5,NF2,ITGB4,ITGA3,LEF1 | | 3.40E+00 | | 7.80E-09 | | 7.51E-06 | |
| GO:0003382 | epithelial cell morphogenesis | 76 | 78 | | 5 | | 219 | | 2.23 | | 71 | | 207253 | | 0.03 | | EPB41L5,CDH1,PLOD3,CGN,MET | | 4.20E+00 | | 2.43E-08 | | 2.11E-05 | |
| GO:0042063 | gliogenesis | 281 | 295 | | 7 | | 217 | | 3.12 | | 274 | | 207050 | | 0.13 | | SOX5,PTPRJ,NF2,TLR2,EIF2B5,DUSP10,LEF1 | | 3.19E+00 | | 3.08E-08 | | 2.42E-05 | |
| GO:0061138 | morphogenesis of a branching epithelium | 300 | 307 | | 7 | | 217 | | 3.12 | | 293 | | 207031 | | 0.14 | | DLG1,PDGFA,SPINT1,CDH1,SLC12A2,MET,LEF1 | | 3.13E+00 | | 4.80E-08 | | 2.60E-05 | |
| GO:0002064 | epithelial cell development | 295 | 298 | | 7 | | 217 | | 3.12 | | 288 | | 207036 | | 0.14 | | TFAP2A,EPB41L5,S1PR3,CDH1,PLOD3,CGN,MET | | 3.14E+00 | | 4.28E-08 | | 2.60E-05 | |
| GO:0006914 | autophagy | 448 | 464 | | 8 | | 216 | | 3.57 | | 440 | | 206884 | | 0.21 | | CISD2,S100A8,UVRAG,ACBD5,RAB12,ITGB4,KIAA1324,ATG9A | | 2.86E+00 | | 4.08E-08 | | 2.60E-05 | |
| GO:0030111 | regulation of Wnt signaling pathway | 451 | 488 | | 8 | | 216 | | 3.57 | | 443 | | 206881 | | 0.21 | | ZRANB1,CDH1,JUP,ITGA3,TLR2,CTNND1,SULF2,LEF1 | | 2.85E+00 | | 4.30E-08 | | 2.60E-05 | |
| GO:0007369 | gastrulation | 300 | 310 | | 7 | | 217 | | 3.12 | | 293 | | 207031 | | 0.14 | | SNAI1,EPB41L5,JUP,NF2,ITGB4,ITGA3,LEF1 | | 3.13E+00 | | 4.80E-08 | | 2.60E-05 | |
| GO:0086001 | cardiac muscle cell action potential | 95 | 100 | | 5 | | 219 | | 2.23 | | 90 | | 207234 | | 0.04 | | DSG2,DSC2,DSP,JUP,ATP1B1 | | 3.96E+00 | | 7.50E-08 | | 3.18E-05 | |
| GO:0086005 | ventricular cardiac muscle cell action potential | 37 | 41 | | 4 | | 220 | | 1.79 | | 33 | | 207291 | | 0.02 | | DSG2,DSC2,DSP,JUP | | 4.74E+00 | | 8.48E-08 | | 3.18E-05 | |
| GO:0086065 | cell communication involved in cardiac conduction | 98 | 103 | | 5 | | 219 | | 2.23 | | 93 | | 207231 | | 0.04 | | DSG2,DSC2,DSP,JUP,ATP1B1 | | 3.93E+00 | | 8.76E-08 | | 3.18E-05 | |
| GO:0072001 | renal system development | 479 | 499 | | 8 | | 216 | | 3.57 | | 471 | | 206853 | | 0.23 | | DLG1,TFAP2A,PROM1,PDGFA,ITGB4,ITGA3,ITGA6,SULF2 | | 2.79E+00 | | 6.81E-08 | | 3.18E-05 | |
| GO:0001701 | in utero embryonic development | 481 | 485 | | 8 | | 216 | | 3.57 | | 473 | | 206851 | | 0.23 | | SNAI1,EPB41L5,RRN3,SPINT1,CDH1,SOX5,PLOD3,LEF1 | | 2.78E+00 | | 7.02E-08 | | 3.18E-05 | |
| GO:0001763 | morphogenesis of a branching structure | 328 | 335 | | 7 | | 217 | | 3.12 | | 321 | | 207003 | | 0.15 | | DLG1,PDGFA,SPINT1,CDH1,SLC12A2,MET,LEF1 | | 3.04E+00 | | 8.79E-08 | | 3.18E-05 | |
| GO:0048709 | oligodendrocyte differentiation | 97 | 97 | | 5 | | 219 | | 2.23 | | 92 | | 207232 | | 0.04 | | SOX5,PTPRJ,TLR2,EIF2B5,DUSP10 | | 3.94E+00 | | 8.32E-08 | | 3.18E-05 | |
| GO:0060541 | respiratory system development | 319 | 325 | | 7 | | 217 | | 3.12 | | 312 | | 207012 | | 0.15 | | PDGFA,PLOD3,EIF4EBP1,ITGA3,DHCR7,FOXP4,LEF1 | | 3.06E+00 | | 7.28E-08 | | 3.18E-05 | |
| GO:0045785 | positive regulation of cell adhesion | 345 | 350 | | 7 | | 217 | | 3.12 | | 338 | | 206986 | | 0.16 | | EPB41L5,NET1,PTPRJ,CX3CL1,ITGA3,ITGA6,LEF1 | | 2.98E+00 | | 1.24E-07 | | 4.29E-05 | |
| GO:0001704 | formation of primary germ layer | 208 | 216 | | 6 | | 218 | | 2.68 | | 202 | | 207122 | | 0.1 | | SNAI1,EPB41L5,NF2,ITGB4,ITGA3,LEF1 | | 3.34E+00 | | 1.29E-07 | | 4.30E-05 | |
| GO:0022407 | regulation of cell-cell adhesion | 217 | 232 | | 6 | | 218 | | 2.68 | | 211 | | 207113 | | 0.1 | | EPB41L5,CDH1,CX3CL1,NF2,ITGA6,LEF1 | | 3.30E+00 | | 1.65E-07 | | 5.31E-05 | |
| GO:2000736 | regulation of stem cell differentiation | 221 | 233 | | 6 | | 218 | | 2.68 | | 215 | | 207109 | | 0.1 | | SNAI1,EFNA1,EPB41L5,SOX5,JUP,LEF1 | | 3.28E+00 | | 1.84E-07 | | 5.70E-05 | |
| GO:0010001 | glial cell differentiation | 246 | 250 | | 6 | | 218 | | 2.68 | | 240 | | 207084 | | 0.12 | | SOX5,PTPRJ,TLR2,EIF2B5,DUSP10,LEF1 | | 3.17E+00 | | 3.45E-07 | | 9.99E-05 | |
| GO:0061337 | cardiac conduction | 129 | 134 | | 5 | | 219 | | 2.23 | | 124 | | 207200 | | 0.06 | | DSG2,DSC2,DSP,JUP,ATP1B1 | | 3.64E+00 | | 3.46E-07 | | 9.99E-05 | |
| GO:0086091 | regulation of heart rate by cardiac conduction | 53 | 57 | | 4 | | 220 | | 1.79 | | 49 | | 207275 | | 0.02 | | DSG2,DSC2,DSP,JUP | | 4.34E+00 | | 3.71E-07 | | 1.04E-04 | |
| GO:0086002 | cardiac muscle cell action potential involved in contraction | 55 | 59 | | 4 | | 220 | | 1.79 | | 51 | | 207273 | | 0.02 | | DSG2,DSC2,DSP,JUP | | 4.30E+00 | | 4.31E-07 | | 1.13E-04 | |
| GO:0097305 | response to alcohol | 415 | 418 | | 7 | | 217 | | 3.12 | | 408 | | 206916 | | 0.2 | | PDGFA,CCNA2,S100A8,CDH1,DEFB1,EIF4EBP1,JUP | | 2.79E+00 | | 4.27E-07 | | 1.13E-04 | |
| GO:1901617 | organic hydroxy compound biosynthetic process | 418 | 424 | | 7 | | 217 | | 3.12 | | 411 | | 206913 | | 0.2 | | SNAI1,BBOX1,LPCAT2,DHCR7,CYB5R1,SLC44A2,LCAT | | 2.79E+00 | | 4.48E-07 | | 1.14E-04 | |
| GO:0034332 | adherens junction organization | 259 | 291 | | 6 | | 218 | | 2.68 | | 253 | | 207071 | | 0.12 | | EPB41L5,CDH1,PTPRJ,DSP,JUP,CTNND1 | | 3.11E+00 | | 4.65E-07 | | 1.15E-04 | |
| GO:0060693 | regulation of branching involved in salivary gland morphogenesis | 15 | 16 | | 3 | | 221 | | 1.34 | | 12 | | 207312 | | 0.01 | | PDGFA,CDH1,MET | | 5.46E+00 | | 5.59E-07 | | 1.35E-04 | |
| GO:2000027 | regulation of organ morphogenesis | 283 | 291 | | 6 | | 218 | | 2.68 | | 277 | | 207047 | | 0.13 | | TFAP2A,PDGFA,DVL3,CDH1,JUP,MET | | 3.02E+00 | | 7.79E-07 | | 1.81E-04 | |
| GO:0030324 | lung development | 284 | 286 | | 6 | | 218 | | 2.68 | | 278 | | 207046 | | 0.13 | | PDGFA,PLOD3,EIF4EBP1,ITGA3,DHCR7,FOXP4 | | 3.02E+00 | | 7.95E-07 | | 1.81E-04 | |
| GO:0030323 | respiratory tube development | 289 | 291 | | 6 | | 218 | | 2.68 | | 283 | | 207041 | | 0.14 | | PDGFA,PLOD3,EIF4EBP1,ITGA3,DHCR7,FOXP4 | | 3.00E+00 | | 8.80E-07 | | 1.96E-04 | |
| GO:0043409 | negative regulation of MAPK cascade | 301 | 305 | | 6 | | 218 | | 2.68 | | 295 | | 207029 | | 0.14 | | SLC9A3R1,PTPRJ,NF2,NUP62,FOXM1,DUSP10 | | 2.96E+00 | | 1.11E-06 | | 2.30E-04 | |
| GO:0010810 | regulation of cell-substrate adhesion | 300 | 311 | | 6 | | 218 | | 2.68 | | 294 | | 207030 | | 0.14 | | EPB41L5,NET1,PTPRJ,NF2,ITGA3,ITGA6 | | 2.96E+00 | | 1.09E-06 | | 2.30E-04 | |
| GO:0022612 | gland morphogenesis | 162 | 163 | | 5 | | 219 | | 2.23 | | 157 | | 207167 | | 0.08 | | PDGFA,NET1,CDH1,SLC12A2,MET | | 3.41E+00 | | 1.07E-06 | | 2.30E-04 | |
| GO:0055117 | regulation of cardiac muscle contraction | 168 | 173 | | 5 | | 219 | | 2.23 | | 163 | | 207161 | | 0.08 | | DSG2,DSC2,DSP,JUP,ATP1B1 | | 3.37E+00 | | 1.27E-06 | | 2.57E-04 | |
| GO:0042476 | odontogenesis | 172 | 183 | | 5 | | 219 | | 2.23 | | 167 | | 207157 | | 0.08 | | TFAP2A,NF2,ITGB4,ITGA6,LEF1 | | 3.34E+00 | | 1.43E-06 | | 2.82E-04 | |
| GO:0010811 | positive regulation of cell-substrate adhesion | 174 | 175 | | 5 | | 219 | | 2.23 | | 169 | | 207155 | | 0.08 | | EPB41L5,NET1,PTPRJ,ITGA3,ITGA6 | | 3.33E+00 | | 1.51E-06 | | 2.92E-04 | |
| GO:0086004 | regulation of cardiac muscle cell contraction | 78 | 82 | | 4 | | 220 | | 1.79 | | 74 | | 207250 | | 0.04 | | DSG2,DSC2,DSP,JUP | | 3.93E+00 | | 1.77E-06 | | 3.31E-04 | |
| GO:0046165 | alcohol biosynthetic process | 327 | 330 | | 6 | | 218 | | 2.68 | | 321 | | 207003 | | 0.15 | | SNAI1,LPCAT2,DHCR7,CYB5R1,SLC44A2,LCAT | | 2.88E+00 | | 1.80E-06 | | 3.31E-04 | |
| GO:0008016 | regulation of heart contraction | 335 | 343 | | 6 | | 218 | | 2.68 | | 329 | | 206995 | | 0.16 | | DSG2,DSC2,S100A1,DSP,JUP,ATP1B1 | | 2.85E+00 | | 2.06E-06 | | 3.73E-04 | |
| GO:1903115 | regulation of actin filament-based movement | 82 | 86 | | 4 | | 220 | | 1.79 | | 78 | | 207246 | | 0.04 | | DSG2,DSC2,DSP,JUP | | 3.88E+00 | | 2.16E-06 | | 3.83E-04 | |
| GO:0035637 | multicellular organismal signaling | 341 | 346 | | 6 | | 218 | | 2.68 | | 335 | | 206989 | | 0.16 | | DSG2,DSC2,DSP,JUP,ATP1B1,EIF2B5 | | 2.83E+00 | | 2.28E-06 | | 3.96E-04 | |
| GO:0006942 | regulation of striated muscle contraction | 193 | 198 | | 5 | | 219 | | 2.23 | | 188 | | 207136 | | 0.09 | | DSG2,DSC2,DSP,JUP,ATP1B1 | | 3.23E+00 | | 2.51E-06 | | 4.27E-04 | |
| GO:0060047 | heart contraction | 353 | 361 | | 6 | | 218 | | 2.68 | | 347 | | 206977 | | 0.17 | | DSG2,DSC2,S100A1,DSP,JUP,ATP1B1 | | 2.80E+00 | | 2.79E-06 | | 4.64E-04 | |
| GO:0003015 | heart process | 356 | 364 | | 6 | | 218 | | 2.68 | | 350 | | 206974 | | 0.17 | | DSG2,DSC2,S100A1,DSP,JUP,ATP1B1 | | 2.79E+00 | | 2.92E-06 | | 4.69E-04 | |
| GO:0048565 | digestive tract development | 199 | 206 | | 5 | | 219 | | 2.23 | | 194 | | 207130 | | 0.09 | | EPB41L5,CDH1,ITGB4,ITGA6,FOXP4 | | 3.19E+00 | | 2.92E-06 | | 4.69E-04 | |
| GO:0050830 | defense response to Gram-positive bacterium | 95 | 95 | | 4 | | 220 | | 1.79 | | 91 | | 207233 | | 0.04 | | MMP7,DEFB1,HIST1H2BK,TLR2 | | 3.72E+00 | | 3.89E-06 | | 6.03E-04 | |
| GO:0055123 | digestive system development | 211 | 218 | | 5 | | 219 | | 2.23 | | 206 | | 207118 | | 0.1 | | EPB41L5,CDH1,ITGB4,ITGA6,FOXP4 | | 3.13E+00 | | 3.88E-06 | | 6.03E-04 | |
| GO:2001234 | negative regulation of apoptotic signaling pathway | 381 | 386 | | 6 | | 218 | | 2.68 | | 375 | | 206949 | | 0.18 | | SNAI1,RAF1,RRN3,CX3CL1,ITGA6,KIAA1324 | | 2.72E+00 | | 4.31E-06 | | 6.55E-04 | |
| GO:0006921 | cellular component disassembly involved in execution phase of apoptosis | 220 | 224 | | 5 | | 219 | | 2.23 | | 215 | | 207109 | | 0.1 | | DSG2,CDH1,OCLN,DSP,TOP2A | | 3.09E+00 | | 4.76E-06 | | 7.11E-04 | |
| GO:0042475 | odontogenesis of dentin-containing tooth | 102 | 109 | | 4 | | 220 | | 1.79 | | 98 | | 207226 | | 0.05 | | NF2,ITGB4,ITGA6,LEF1 | | 3.65E+00 | | 5.17E-06 | | 7.46E-04 | |
| GO:0022408 | negative regulation of cell-cell adhesion | 102 | 117 | | 4 | | 220 | | 1.79 | | 98 | | 207226 | | 0.05 | | EPB41L5,CDH1,NF2,LEF1 | | 3.65E+00 | | 5.17E-06 | | 7.46E-04 | |
| GO:0001508 | action potential | 401 | 406 | | 6 | | 218 | | 2.68 | | 395 | | 206929 | | 0.19 | | DSG2,DSC2,DSP,JUP,ATP1B1,EIF2B5 | | 2.67E+00 | | 5.76E-06 | | 8.19E-04 | |
| GO:0048839 | inner ear development | 230 | 235 | | 5 | | 219 | | 2.23 | | 225 | | 207099 | | 0.11 | | TFAP2A,PDGFA,DVL3,NET1,CDH1 | | 3.05E+00 | | 5.90E-06 | | 8.25E-04 | |
| GO:0032103 | positive regulation of response to external stimulus | 412 | 415 | | 6 | | 218 | | 2.68 | | 406 | | 206918 | | 0.2 | | S100A8,PTPRJ,CX3CL1,MET,RAB12,TLR2 | | 2.64E+00 | | 6.72E-06 | | 9.25E-04 | |
| GO:0010717 | regulation of epithelial to mesenchymal transition | 113 | 118 | | 4 | | 220 | | 1.79 | | 109 | | 207215 | | 0.05 | | SNAI1,EFNA1,EPB41L5,LEF1 | | 3.54E+00 | | 7.75E-06 | | 1.05E-03 | |
| GO:0030307 | positive regulation of cell growth | 247 | 252 | | 5 | | 219 | | 2.23 | | 242 | | 207082 | | 0.12 | | NET1,S100A8,EXOSC4,TNFRSF12A,LEF1 | | 2.97E+00 | | 8.33E-06 | | 1.11E-03 | |
| GO:0043954 | cellular component maintenance | 36 | 36 | | 3 | | 221 | | 1.34 | | 33 | | 207291 | | 0.02 | | PLEKHA7,TANC1,ITGA3 | | 4.45E+00 | | 8.63E-06 | | 1.13E-03 | |
| GO:0001822 | kidney development | 449 | 462 | | 6 | | 218 | | 2.68 | | 443 | | 206881 | | 0.21 | | DLG1,TFAP2A,PROM1,PDGFA,ITGA3,SULF2 | | 2.55E+00 | | 1.09E-05 | | 1.39E-03 | |
| GO:0030879 | mammary gland development | 261 | 265 | | 5 | | 219 | | 2.23 | | 256 | | 207068 | | 0.12 | | NET1,SLC29A1,HK2,SLC12A2,LEF1 | | 2.92E+00 | | 1.09E-05 | | 1.39E-03 | |
| GO:0060445 | branching involved in salivary gland morphogenesis | 40 | 41 | | 3 | | 221 | | 1.34 | | 37 | | 207287 | | 0.02 | | PDGFA,CDH1,MET | | 4.33E+00 | | 1.19E-05 | | 1.50E-03 | |
| GO:0007179 | transforming growth factor beta receptor signaling pathway | 458 | 462 | | 6 | | 218 | | 2.68 | | 452 | | 206872 | | 0.22 | | PDGFA,F11R,SMURF1,ATL2,CGN,ITGA3 | | 2.53E+00 | | 1.22E-05 | | 1.51E-03 | |
| GO:0014031 | mesenchymal cell development | 269 | 274 | | 5 | | 219 | | 2.23 | | 264 | | 207060 | | 0.13 | | SNAI1,TFAP2A,EFNA1,EPB41L5,LEF1 | | 2.89E+00 | | 1.26E-05 | | 1.53E-03 | |
| GO:0010720 | positive regulation of cell development | 462 | 467 | | 6 | | 218 | | 2.68 | | 456 | | 206868 | | 0.22 | | SNAI1,EPB41L5,NET1,TLR2,TNFRSF12A,LEF1 | | 2.52E+00 | | 1.28E-05 | | 1.54E-03 | |
| GO:0010770 | positive regulation of cell morphogenesis involved in differentiation | 131 | 135 | | 4 | | 220 | | 1.79 | | 127 | | 207197 | | 0.06 | | SNAI1,EPB41L5,NET1,LEF1 | | 3.39E+00 | | 1.39E-05 | | 1.59E-03 | |
| GO:0048864 | stem cell development | 469 | 474 | | 6 | | 218 | | 2.68 | | 463 | | 206861 | | 0.22 | | SNAI1,TFAP2A,EFNA1,RAF1,EPB41L5,LEF1 | | 2.51E+00 | | 1.40E-05 | | 1.59E-03 | |
| GO:0008544 | epidermis development | 468 | 470 | | 6 | | 218 | | 2.68 | | 462 | | 206862 | | 0.22 | | SNAI1,TFAP2A,PDGFA,ST14,PLOD3,DSP | | 2.51E+00 | | 1.38E-05 | | 1.59E-03 | |
| GO:0060021 | palate development | 130 | 138 | | 4 | | 220 | | 1.79 | | 126 | | 207198 | | 0.06 | | DLG1,SNAI1,TFAP2A,LEF1 | | 3.40E+00 | | 1.35E-05 | | 1.59E-03 | |
| GO:0043583 | ear development | 276 | 281 | | 5 | | 219 | | 2.23 | | 271 | | 207053 | | 0.13 | | TFAP2A,PDGFA,DVL3,NET1,CDH1 | | 2.86E+00 | | 1.42E-05 | | 1.60E-03 | |
| GO:0007009 | plasma membrane organization | 472 | 485 | | 6 | | 218 | | 2.68 | | 466 | | 206858 | | 0.22 | | DLG1,CDH1,JUP,ATP1B1,ITGA3,BAIAP2L1 | | 2.50E+00 | | 1.45E-05 | | 1.61E-03 | |
| GO:0007162 | negative regulation of cell adhesion | 284 | 299 | | 5 | | 219 | | 2.23 | | 279 | | 207045 | | 0.13 | | EPB41L5,CDH1,TGFBI,NF2,LEF1 | | 2.83E+00 | | 1.63E-05 | | 1.77E-03 | |
| GO:0007034 | vacuolar transport | 136 | 137 | | 4 | | 220 | | 1.79 | | 132 | | 207192 | | 0.06 | | HOOK1,SORT1,CHMP4C,RAB12 | | 3.35E+00 | | 1.61E-05 | | 1.77E-03 | |
| GO:0051656 | establishment of organelle localization | 485 | 497 | | 6 | | 218 | | 2.68 | | 479 | | 206845 | | 0.23 | | CENPF,NET1,SPIRE1,DLGAP5,YKT6,NUSAP1 | | 2.48E+00 | | 1.68E-05 | | 1.80E-03 | |
| GO:0002027 | regulation of heart rate | 141 | 145 | | 4 | | 220 | | 1.79 | | 137 | | 207187 | | 0.07 | | DSG2,DSC2,DSP,JUP | | 3.31E+00 | | 1.86E-05 | | 1.96E-03 | |
| GO:0097194 | execution phase of apoptosis | 299 | 303 | | 5 | | 219 | | 2.23 | | 294 | | 207030 | | 0.14 | | DSG2,CDH1,OCLN,DSP,TOP2A | | 2.78E+00 | | 2.08E-05 | | 2.18E-03 | |
| GO:0048762 | mesenchymal cell differentiation | 304 | 309 | | 5 | | 219 | | 2.23 | | 299 | | 207025 | | 0.14 | | SNAI1,TFAP2A,EFNA1,EPB41L5,LEF1 | | 2.76E+00 | | 2.25E-05 | | 2.33E-03 | |
| GO:1901654 | response to ketone | 154 | 154 | | 4 | | 220 | | 1.79 | | 150 | | 207174 | | 0.07 | | DCPS,DSG2,DEFB1,TLR2 | | 3.22E+00 | | 2.62E-05 | | 2.63E-03 | |
| GO:0006937 | regulation of muscle contraction | 313 | 318 | | 5 | | 219 | | 2.23 | | 308 | | 207016 | | 0.15 | | DSG2,DSC2,DSP,JUP,ATP1B1 | | 2.73E+00 | | 2.59E-05 | | 2.63E-03 | |
| GO:0048730 | epidermis morphogenesis | 52 | 52 | | 3 | | 221 | | 1.34 | | 49 | | 207275 | | 0.02 | | SNAI1,TFAP2A,PLOD3 | | 4.05E+00 | | 2.64E-05 | | 2.63E-03 | |
| GO:0046470 | phosphatidylcholine metabolic process | 155 | 157 | | 4 | | 220 | | 1.79 | | 151 | | 207173 | | 0.07 | | MBOAT2,LPCAT2,SLC44A2,LCAT | | 3.22E+00 | | 2.69E-05 | | 2.65E-03 | |
| GO:0007435 | salivary gland morphogenesis | 53 | 54 | | 3 | | 221 | | 1.34 | | 50 | | 207274 | | 0.02 | | PDGFA,CDH1,MET | | 4.03E+00 | | 2.79E-05 | | 2.72E-03 | |
| GO:0007088 | regulation of mitotic nuclear division | 320 | 327 | | 5 | | 219 | | 2.23 | | 315 | | 207009 | | 0.15 | | CENPF,BUB1B,DLGAP5,TOM1L1,NUSAP1 | | 2.71E+00 | | 2.88E-05 | | 2.77E-03 | |
| GO:0042742 | defense response to bacterium | 324 | 325 | | 5 | | 219 | | 2.23 | | 319 | | 207005 | | 0.15 | | MMP7,S100A8,DEFB1,HIST1H2BK,TLR2 | | 2.70E+00 | | 3.05E-05 | | 2.91E-03 | |
| GO:0035434 | copper ion transmembrane transport | 8 | 8 | | 2 | | 222 | | 0.89 | | 6 | | 207318 | | 0 | | SLC31A2,SLC31A1 | | 5.74E+00 | | 3.23E-05 | | 3.01E-03 | |
| GO:0007431 | salivary gland development | 56 | 57 | | 3 | | 221 | | 1.34 | | 53 | | 207271 | | 0.03 | | PDGFA,CDH1,MET | | 3.97E+00 | | 3.30E-05 | | 3.01E-03 | |
| GO:0019731 | antibacterial humoral response | 56 | 56 | | 3 | | 221 | | 1.34 | | 53 | | 207271 | | 0.03 | | MMP7,DEFB1,HIST1H2BK | | 3.97E+00 | | 3.30E-05 | | 3.01E-03 | |
| GO:0035878 | nail development | 8 | 15 | | 2 | | 222 | | 0.89 | | 6 | | 207318 | | 0 | | ITGB4,ITGA6 | | 5.74E+00 | | 3.23E-05 | | 3.01E-03 | |
| GO:0072006 | nephron development | 165 | 173 | | 4 | | 220 | | 1.79 | | 161 | | 207163 | | 0.08 | | TFAP2A,PROM1,ITGA3,SULF2 | | 3.15E+00 | | 3.43E-05 | | 3.10E-03 | |
| GO:0008333 | endosome to lysosome transport | 57 | 58 | | 3 | | 221 | | 1.34 | | 54 | | 207270 | | 0.03 | | HOOK1,SORT1,RAB12 | | 3.95E+00 | | 3.48E-05 | | 3.11E-03 | |
| GO:0051898 | negative regulation of protein kinase B signaling | 58 | 60 | | 3 | | 221 | | 1.34 | | 55 | | 207269 | | 0.03 | | SLC9A3R1,DLG1,PTPRJ | | 3.93E+00 | | 3.66E-05 | | 3.24E-03 | |
| GO:0072659 | protein localization to plasma membrane | 344 | 357 | | 5 | | 219 | | 2.23 | | 339 | | 206985 | | 0.16 | | DLG1,CDH1,JUP,ATP1B1,ITGA3 | | 2.63E+00 | | 4.06E-05 | | 3.52E-03 | |
| GO:0010718 | positive regulation of epithelial to mesenchymal transition | 60 | 64 | | 3 | | 221 | | 1.34 | | 57 | | 207267 | | 0.03 | | SNAI1,EPB41L5,LEF1 | | 3.90E+00 | | 4.06E-05 | | 3.52E-03 | |
| GO:0019730 | antimicrobial humoral response | 61 | 61 | | 3 | | 221 | | 1.34 | | 58 | | 207266 | | 0.03 | | MMP7,DEFB1,HIST1H2BK | | 3.88E+00 | | 4.26E-05 | | 3.66E-03 | |
| GO:0045927 | positive regulation of growth | 353 | 358 | | 5 | | 219 | | 2.23 | | 348 | | 206976 | | 0.17 | | NET1,S100A8,EXOSC4,TNFRSF12A,LEF1 | | 2.61E+00 | | 4.58E-05 | | 3.89E-03 | |
| GO:0051783 | regulation of nuclear division | 354 | 361 | | 5 | | 219 | | 2.23 | | 349 | | 206975 | | 0.17 | | CENPF,BUB1B,DLGAP5,TOM1L1,NUSAP1 | | 2.61E+00 | | 4.64E-05 | | 3.91E-03 | |
| GO:0030177 | positive regulation of Wnt signaling pathway | 179 | 183 | | 4 | | 220 | | 1.79 | | 175 | | 207149 | | 0.08 | | ZRANB1,JUP,TLR2,SULF2 | | 3.07E+00 | | 4.71E-05 | | 3.93E-03 | |
| GO:0043589 | skin morphogenesis | 64 | 64 | | 3 | | 221 | | 1.34 | | 61 | | 207263 | | 0.03 | | SNAI1,TFAP2A,PLOD3 | | 3.83E+00 | | 4.92E-05 | | 4.06E-03 | |
| GO:0002934 | desmosome organization | 10 | 13 | | 2 | | 222 | | 0.89 | | 8 | | 207316 | | 0 | | DSP,JUP | | 5.45E+00 | | 5.19E-05 | | 4.24E-03 | |
| GO:0042439 | ethanolamine-containing compound metabolic process | 190 | 192 | | 4 | | 220 | | 1.79 | | 186 | | 207138 | | 0.09 | | MBOAT2,LPCAT2,SLC44A2,LCAT | | 3.01E+00 | | 5.94E-05 | | 4.72E-03 | |
| GO:0001837 | epithelial to mesenchymal transition | 190 | 195 | | 4 | | 220 | | 1.79 | | 186 | | 207138 | | 0.09 | | SNAI1,EFNA1,EPB41L5,LEF1 | | 3.01E+00 | | 5.94E-05 | | 4.72E-03 | |
| GO:2001236 | regulation of extrinsic apoptotic signaling pathway | 373 | 379 | | 5 | | 219 | | 2.23 | | 368 | | 206956 | | 0.18 | | RAF1,CX3CL1,ITGA6,KIAA1324,TNFRSF12A | | 2.55E+00 | | 5.94E-05 | | 4.72E-03 | |
| GO:0031668 | cellular response to extracellular stimulus | 374 | 379 | | 5 | | 219 | | 2.23 | | 369 | | 206955 | | 0.18 | | DSC2,RAB12,ITGA6,KIAA1324,ATG9A | | 2.55E+00 | | 6.02E-05 | | 4.74E-03 | |
| GO:0007044 | cell-substrate junction assembly | 194 | 204 | | 4 | | 220 | | 1.79 | | 190 | | 207134 | | 0.09 | | EPB41L5,PTPRJ,ITGB4,ITGA6 | | 2.99E+00 | | 6.43E-05 | | 4.97E-03 | |
| GO:0090257 | regulation of muscle system process | 380 | 385 | | 5 | | 219 | | 2.23 | | 375 | | 206949 | | 0.18 | | DSG2,DSC2,DSP,JUP,ATP1B1 | | 2.53E+00 | | 6.48E-05 | | 4.97E-03 | |
| GO:0035272 | exocrine system development | 70 | 71 | | 3 | | 221 | | 1.34 | | 67 | | 207257 | | 0.03 | | PDGFA,CDH1,MET | | 3.74E+00 | | 6.44E-05 | | 4.97E-03 | |
